# Game Intelligence in Team Sports

**DOI:** 10.1371/journal.pone.0125453

**Published:** 2015-05-13

**Authors:** Jan Lennartsson, Nicklas Lidström, Carl Lindberg

**Affiliations:** 1 Chalmers University of Technology and Gothenburg University, Sweden; 2 The Second Swedish National Pension Fund and Uppsala University, Sweden; 3 Independent Researcher, Vasteras, Sweden; University of the Basque Country, SPAIN

## Abstract

We set up a game theoretic framework to analyze a wide range of situations from team sports. A fundamental idea is the concept of *potential*; the probability of the offense scoring the next goal minus the probability that the next goal is made by the defense. We develop categorical as well as continuous models, and obtain optimal strategies for both offense and defense. A main result is that the optimal defensive strategy is to minimize the maximum potential of all offensive strategies.

## Introduction

A subset of all team sports is the ones where two opposing teams each have a goal to defend, and the team which scores the most points win the game. This paper analyzes general situation tactics in such sports, henceforth denoted *team sports*. Game intelligence in team sports is usually regarded as something very incomprehensible, and excellent players are often praised for how they “read the game”. Even though most would agree on what constitutes good skills—technique, strength, agility, endurance, etc—it is less obvious what characterizes a good player in terms of game intelligence.

We will make an attempt at analyzing the concept of game intelligence from a game theoretic perspective. To this end, we assume that a player’s overall ability can be categorized into two parts. First, the ability to decide on a strategy, which is in some sense optimal, in each encountered game situation. Second, to carry out the chosen strategy. The first category is what typically is contained in the concept of game intelligence, while the last category has to do with a player’s skill set. The present paper will focus on the first category. Obviously, one can never know which choice would have worked out the best on each particular occasion. However, we show in this paper that we can find strategies which are optimal in the mean. To analyze such situations, we adopt a game theoretic framework. We will model game situations as so called zero-sum games, i e games where the players have exactly opposite rewards. In our setting, a goal made by the offense has value one, which is exactly the negative of the value it has for the defense. Conversely, a goal made by the defense has the value minus one for the offense which again is exactly the negative of the value it has for the defense. Further, we associate a utility to each player’s choices. We will define the utility function for team sports to be the *potential*; the probability of the attacking team scoring the next goal minus the probability that the next goal is made by the defending team. Given this setup, we can model a variety of team sport game situations, and solve these using standard game theoretic methods. An implication of our approach is that game intelligence is not something incomprehensible, but rather an acquired skill. Note that our setup is different than the so-called potential games in e g [1]. We have chosen to denote the utility function by potential because of its natural interpretation as a scalar potential for a conservative vector field.

The first part of the paper gives the theoretical foundation underlying our analysis, including the concept of potential fields, and derives some results with applications to game intelligence. The following sections focus on game situations where the players make decisions based on a given set of strategies. Here, we apply principles from game theory to determine which decisions are optimal. A main consequence of our problem set up is that the optimal defensive strategy is to make the best offensive choice, in terms of potential, as bad as possible. Further, the optimal strategy for the offense is to distribute shots between the players so that, for every player, a shot should be taken if and only if the potential is larger than a certain threshold. This threshold is the same for all players. It is important to note that the optimal strategy does not guarantee a successful outcome on each occasion. Rather, the optimal strategy for a specific situation gives the best outcome in terms of potential.

There is an extensive literature on the application of game theory to fixed game situations in sports. In [2], the game theoretic analysis of sports as a quantitative field is introduced by testing if professionals use the mixed Nash equilibrium when they decide on which side, forehand or backhand, to land the first serve. They found that men’s pro tennis players did not randomize their serves in an optimal way, but were rather switching the serve direction too often. Later, [3] found evidence of good strategic play among professionals in the same situation. In the same setting, [4] included in their analysis also the types of court surfaces, and found evidence that servers on faster surfaces tend to hit the serve to an opponent’s backhand too often.

In [5], a game theoretic approach yields that there is room for improvement both in pitch selection in Major League Baseball (MLB) as well as in calling plays in the National Football League (NFL). They can conclude that pitchers throw more fastballs than what is optimal, and that football teams pass less than they ought to. Interestingly, they find in both sports a negative serial correlation in the decisions. Further, [2] find the same result in tennis, and [6] report similar findings for the NFL. This indicates that strategies, in some sports, are changed more often than what we would see if the strategy choices were truly random. In [6], the authors offer the explanation that teams excessively switch play types in order to not be perceived as predictable. The paper [7] present the idea that, in the NFL, the observed temporal dependence in strategy choice is due to that the offense tries to wear down the defense.

In soccer, [8], [9], [10], and [11] all find that during soccer penalty kicks, both the strikers and the goalkeepers are making choices that are consistent with the strategies of Nash equilibria.

Even though fixed game situations play an important role in sports such as ice-hockey, team handball, basketball and soccer, these sports are primarily built up by in-game activity. However, there does not seem to be much literature on quantitative approaches to in-game activity, or to game intelligence which is the scope of the present paper. The papers [12] and [13] are slightly related in spirit to the first part of the present paper. In [12], the performance of an basketball offense is modeled as a network problem. The paper [13] investigates when, in terms of shot quality, a basketball team should shoot. Further, [14] estimate a matching law from basketball scoring data, and are able to verify a good fit. Finally, optimal strategies for an underdog are derived in [15]. In soccer, [16] analyze the in-game decision making of shooting towards the far post as opposed to the near post.

This paper is organized as follows. In Section *Potential*, we introduce the concept of potential, which is of central importance to our analysis. We prove in Section *Fundamental results* a theorem and a lemma. These will be used extensively throughout the paper. Sections *Strategic game situations* and *Extensive game situations* present some standard game theoretic notation and results. Further, we adapt these results to our framework, and give some examples which illustrate the applicability of the theory to game intelligence. In Section *Shot potential*, we define the shot potential as the probability of scoring from a given position, and show how this can be used in the modeling and analysis of various situations in sports. Section *Parameter estimation* presents some possible approaches to estimate the model parameters from data. We conclude with a discussion.

## Potential

We introduce in this section the concept of potential. This idea is fundamental to our analysis, and it is applied in all game situations that we consider.

There are two teams in each game; team A and team B. We define the stochastic expiration time *T* to be the time when the next goal is scored or the game ends. Further, we denote by *V*(*T*) the stochastic variable that takes the value 1 if team A scores, −1 if team B scores, and 0 if no goal is scored before the game is over.

**Definition 1** *The **potential**, v, is defined by*

$$\begin{array}{c} v_{t}={𝔼}_{t}\left[V\left(T\right)\right],\forall t\in \left[0,T\right]. \end{array}$$

Hence, the potential is the probability of team A scoring the next goal, minus the probability that the next goal is made by team B. The potential *v* is a general stochastic process in continuous time which is conditioned on the present states of all players, as well as their respective strategies, skills and tactics. Unfortunately, the potential process *v* is too complex to model explicitly. Hence, we will in this paper analyze *v* only for a set of game situations which are frequently recurring in their specific sports. Given such a situation, we will be able to find the optimal player behavior in that particular setting. If the players adopt the optimal behavior, they improve the potential *v* for all *t*; higher potential from the perspective of team A, and lower from the point of view of team B. Recall that the potential *v* considers not just the probability of scoring, but also the effects of losing ball possession.

To illustrate the concept of potential, we consider a few short examples. In a situation where a team A player has possession with no team B players in her path to the goal, the potential is close to one. In contrast, if team A has ball possession, but there are a large number of skilled and well positioned team B players ahead of them, the potential may be close to zero. Further, a missed pass for team A yields a drop in the potential. This drop could be big for particularly bad passes, possibly down to almost −1.

We are considering *n* categorical alternatives to pursue shot attempts at. Further, the *defensive effort*, *y* ∈ [0, 1]^*n*^, such that ∑*y*_*i*_ = 1, is the proportional effort that team B puts on each of the categorical alternatives in order to reduce the potential of the corresponding shot alternatives. In addition, *x* = (*x*_1_, …, *x*_*n*_) ∈ [0, 1]^*n*^ such that ∑_*i*_ *x*_*i*_ = 1 is the expected proportion of shot opportunities from each offensive alternative *i* = 1, …, *n*. We define the *cumulative potential functions* {*F*_*i*_}_*i* = 1, …, *n*_, for each *i* = 1, …, *n*, as the integral of the corresponding *potential frequency function* *f*_*i*_:[0, 1] × [0, 1] → [−1, 1], such that

$$\begin{array}{c} F_{i}\left(x,y\right)=\int_{0}^{x}f_{i}\left(\xi ,y\right)d\xi , \end{array}$$

for all *x*, *y* ∈ [0, 1]. We show now that the potential frequency function can be understood in terms of the distribution of the quality of the shot opportunities. Consider an offensive alternative *i*. We assume that under normal game conditions, the shot opportunities *Y*_*i*_(*y*) ∈ [−1, 1], given defensive effort *y* in that shot alternative, are drawn randomly and independently from a known distribution *G*_*Y*_*i*_(*y*)_. Here, each value of *Y*_*i*_(*y*) gives the potential for that shot opportunity. The potential frequency function is defined by

$$\begin{array}{c} f_{i}\left(x,y\right)=G_{Y_{i}\left(y\right)}^{-1}\left(1-x\right), \end{array}$$

where (⋅)^−1^ denotes the inverse and *x*, *y* ∈ [0, 1]. Hence, the potential frequency function gives the potential in seeking an additional “infinitesimal” proportion of the shot opportunities at the offensive alternative *i* given expected shot proportion *x* and defense effort *y*. We see that, by construction, the potential frequency functions *f*_*i*_ are monotonically decreasing in the first argument, respectively. This is natural, since a rational offensive approach is to seek shot attempts at the best shot opportunities first and next consider shot opportunities with smaller potential. Further, we will assume that the potential frequency functions are also monotonically decreasing in the second argument, respectively. This models the rational game characteristic that if more defensive effort is put on shot alternative *i*, the potential of that alternative is reduced.

To exemplify, a rational defensive action in response to a high potential categorical offensive alternative *i* would be to increase the defensive effort *y*_*i*_, and decrease some or multiple *y*_*j*_ for *j* ≠ *i*, such that *f*_*i*_(*x*_*i*_, *y*_*i*_) is reduced to the expense of an increase in *f*_*j*_(*x*_*j*_, *y*_*j*_).

We illustrate now the concept of potential with two brief examples, one from ice hockey and one from team handball.

When is it a good idea to break the rules in a way which causes a 2 minute penalty in ice-hockey? Assume that the probability that team A scores during a team B penalty is *p* ∈ (0, 1). Conversely, the probability that team B scores while being one player short is approximately 0. Further, we assume that the potential if team A has scored is 0, as is the potential if no team has scored when the penalty is over. Hence, the potential when the penalty time starts is *p*. This implies that if it is going to be a good decision for a team B player do to something which gives a 2 minute penalty, her action needs to prevent a situation which had a potential higher than *p*.

Consider a wing position in team handball, with index *w*. The wing player shoots when given possession and a free path up to at least *η* meters up from the short side line. The potential of shot opportunities when shooting at exactly *η* meters is *f*_*w*_(*x*_*w*_, *y*_*w*_) under the offensive shot proportions *x* and defensive efforts *y* ∈ [0, 1]^*n*^. If the wing player is supposed to increase the proportion of expected shots, *x*_*w*_, then she needs to take shot opportunities from less than *η* meters. Further, the additional shots will have a lower potential, since it is harder to score from a wide position than from a central one. In addition, if there is an increased defensive effort put on the wing player, i e if *y*_*w*_ increases, then she needs to lower the threshold *η* in order to maintain the same shot proportion *x*_*w*_.

**Remark 2** *Note that it is straightforward to extend the concept of potential to let it account for various penalties or to sports where a goal can have different value in points depending on how it was made. Basketball is one example of such a sport.*

## Fundamental results

Here we state and prove two results, from which a lot of interesting conclusions are drawn.

Consider an offense categorized into *n* alternatives. We now state the following theorem, where *A* ^*C*^ denotes the complement of the set *A*:

**Theorem 3 (Tactical Theorem of Team Sports)** *Suppose that the potential frequency functions {f_i_(x, y)}_i = 1, …, n_, for x, y ∈ [0, 1] are continuously differentiable and decreasing in x and y, respectively, and that $\frac{\partial^{2}}{\partial y^{2}}f_{i}(x,y)$ is non-negative and continuous. Assume that we are given a fixed defensive effort y ∈* [0, 1]^*n*^ *such that* $\sum_{i=1}^{n}y_{i}=1$. *Further, the expected shot proportions* x* ∈ [0, 1]^*n*^, *subject to* $\sum_{i=1}^{n}x_{i}^{*}=1$, *satisfies*

$$\begin{array}{c} f_{j}\left(x_{j}^{*},y_{j}\right)=f_{k}\left(x_{k}^{*},y_{k}\right), \end{array}$$ (1)

*for all j, k in some subset* 𝓚_*o*_(x*) ⊂ [1, …, *n*], *with* $x_{i}^{*}>0$ if and only if i ∈ 𝓚_o_(x*), for $\sum_{j\in {𝓚}_{o}}x_{j}^{*}=1$, *and where* $f_{i}(0,y_{i})<f_{j}(x_{j}^{*},y_{j})$, *for all* *i* ∈ 𝓚_*o*_(x*)^*C*^ *and any* *j* ∈ 𝓚_*o*_(x*). *Then* x* *maximizes the potential G defined by*

$$\begin{array}{c} G(x,y):=\sum_{i=1}^{n}F_{i}\left(x_{i},y_{i}\right). \end{array}$$ (2)

*Conversely, assume that we are given fixed expected shot proportions* *x* ∈ [0, 1]^*n*^, *with* $\sum_{i=1}^{n}x_{i}=1$, and a defense y*, subject to $\sum_{i=1}^{n}y_{i}^{*}=1$, *which satisfies*

$$\begin{array}{c} \int_{0}^{x_{j}}\frac{\partial f_{j}}{\partial y_{j}}\left(\xi ,y_{j}^{*}\right)d\xi =\int_{0}^{x_{k}}\frac{\partial f_{k}}{\partial y_{k}}\left(\xi ,y_{k}^{*}\right)d\xi \end{array}$$

*for all j, k in some subset* 𝓚_*d*_(y*) ⊂ [1, …, *n*], *with* $y_{i}^{*}>0$ *if and only if i ∈ 𝓚_d_, where* $\sum_{j\in {𝓚}_{d}}y_{j}^{*}=1$. *Further,*

$$\begin{array}{c} \int_{0}^{x_{i}}\frac{\partial f_{i}}{\partial y_{i}}\left(\xi ,0\right)d\xi >\int_{0}^{x_{j}}\frac{\partial f_{j}}{\partial y_{j}}\left(\xi ,y_{j}\right)d\xi \end{array}$$

for all i ∈ 𝓚_d_(y*)^C^ and j ∈ 𝓚_d_(y*). Then y* minimizes the potential G(x, y). Finally, if we can find a point (x*, y*) for which

$$\begin{array}{c} G(x,y^{*})\leq G(x^{*},y^{*})\leq G(x^{*},y), \end{array}$$

for all feasible x, y, i e a point where x* is a maximizer for G when y is fixed at y*, and where y* is a minimizer for G when x is fixed at x*. Then

$$\begin{array}{c} \max_{x}\min_{y}\;G(x,y)=\min_{y}\max_{x}\;G(x,y). \end{array}$$

**Proof**. The first part of the proof follows by noting that a point x* which satisfies the conditions stated in the theorem allows us to apply the Karuch-Kuhn-Tucker optimization principles, see e.g. [17, Lemma 14.5]. This gives us that x* is a local maximum. Further, *G* is concave with respect to *x*, since the *f*_*i*_ are continuously differentiable and decreasing. Hence we have a concave maximization problem, and it follows from [17, Theorem 2.1], that x* is a global maximizer.

The second part of the proof follows completely analogously.

The final conclusion is an immediate consequence of [17, Lemma 14.8].■

Intuitively, it is optimal for the offensive side to always strive to distribute shooting proportions such that all shots which are fired from any position should have a potential larger than some threshold. This threshold is the same for all offensive alternatives. Hence, if the offense plays optimally it should be indifferent to which offensive alternative to assign a small additional shot proportion to. On the contrary, the defensive side should seek to distribute its effort such that if a small defensive effort was added to any defending alternative *i*, these would yield the same decrease in *F*_*i*_.

We give now a simple but important lemma which helps us to understand how the defense should optimally position itself. We give the lemma for a single defender, but it is straightforward to extend it to a multi defender setting.

**Lemma 4 (Positioning for Indifference)** *We assume that the offensive side has n alternatives, each associated with a continuous convex potential function {v_i_(y)}_i = 1, …, n_, of the defensive player’s **position**, location and velocity, y = (y_l_, y_v_). The offense will pursue its best alternative, so the potential of the situation v is defined by*

$$\begin{array}{c} v(y)=\max_{i}v_{i}\left(y\right). \end{array}$$

*For a set 𝓓:* = {*y*: *g*_*i*_(*y*) ≥ 0, ∀*i* = 1, …, *m*} for concave functions g_i_, there exists a global minimizer y* ∈ 𝓓 to v. The point y* is either a minimum to v_i_ for some i = 1, …, n, in which case v_j_(y*) ≤ v_i_(y*) for all j ≠ i, or y* satisfies

$$\begin{array}{c} v_{j}\left(y_{j}^{*}\right)=v_{k}\left(y_{k}^{*}\right) \end{array}$$ (3)

*for all j, k in some subset* 𝓚_*d*_ ⊂ [1, …, *n*], and where v_i_(y*) ≤ v_j_(y*), for all $i\in {𝓚}_{d}^{C}$ and any *j* ∈ 𝓚_*d*_.

**Proof**. The first conclusion follows from [17, Theorem 2.1]. For the second part, if y* is the global minimizer and y* ≠ arg min_*y* ∈ 𝓓_ *v*_*i*_(*y*) for any *i* = 1, …, *n*, then for any *i* such that *v*_*i*_(y*) = *v*(y*) there is a non-zero gradient *q*_*i*_ such that for small *λ* > 0 we have that y* − *λq*_*i*_ ∈ 𝓓 and *v*_*i*_(y* − *λq*_*i*_) < *v*(y*). Further, since y* is the global minimizer and the potential functions are continuous and convex then there exist a *j* ≠ *i* such that *v*(y*) ≤ *v*(y* − *λq*_*i*_) = *v*_*j*_(y* − *λq*_*i*_). Hence, since the potential functions are continuous there exist a *j* ≠ *i* such that *v*_*j*_(y*) = *v*(y*).

We call an offensive alternative *i* *relevant* if *v*_*i*_(y*) = *v*(y*). If there is only a single element in the set of relevant alternatives, that option is called the *dominating* offensive alternative. The lemma above states that given that there is no dominating offensive alternative, the optimal defense position is such that the potential of at least two, possible several, offensive alternatives are equal.

**Remark 5** *Note that in the last parts of the game, the teams may apply a different metric than the potential, such as simply to maximize the probability of scoring, indifferent of the resulting potential of the situation. The Positioning for Indifference lemma obviously works regardless of which underlying functions *v*_*i*_ we choose, as long as they satisfy the conditions of the lemma.*

### Notable examples

In this section, we give some examples of applications to the Tactical Theorem of Team Sports and the Positioning for Indifference lemma. The applications are drawn from ice-hockey and team handball. However, the results above are not at all constrained to these two sports. Rather, the two sports were chosen based on the authors personal sporting backgrounds.

#### Team handball; shot proportions

We will in this example challenge two old “truths” in team handball. These are that an acceptable level of shot efficiency for wing and pivot players are about 80%, while an acceptable corresponding level for backcourt players is about 50%. We have, by the Tactical Theorem of Team Sports, that given a defense *y*, optimal or not, the optimal expected shot proportions x* are such that their potential frequency functions are equal. Intuitively, the best alternative for each offensive player that they do not pursue all have equal potential.

Recall that the potential is the probability of team A scoring the next goal, minus the probability of team B scoring the next goal. Hence, it is different from the probability of team A scoring. In general, the risk of technical faults for reaching a back court or wing shot is less than for a corresponding pass to the pivot, which is more troublesome to set up.

**Fig 1:**
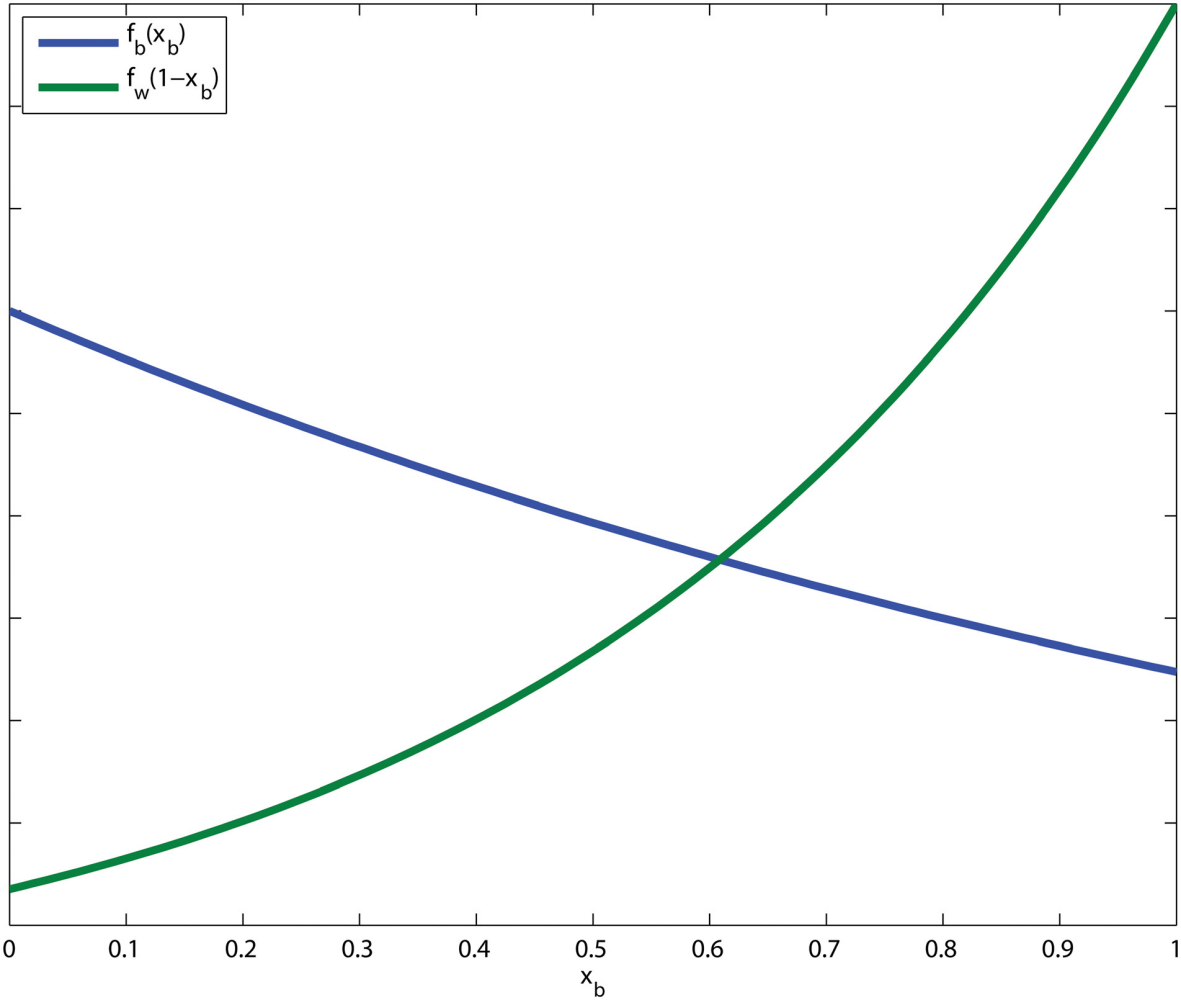
The potential frequency functions *f*_*b*_(*x*_*b*_) and *f*_*w*_(1 − *x*_*b*_).

In order to illustrate the consequence of the Tactical Theorem of Team Sports, we give a simplified example of a situation with only two offensive players; a back court player and a wing player. Suppose, given the current defense, that the potential frequency functions for the back court and wing players are given by *f*_*b*_ and *f*_*w*_, respectively, see Fig 1.

We let *x*_*b*_ denote the proportion of shots fired by the back court player, and thus *x*_*w*_ = 1 − *x*_*b*_ is the proportion of shots fired by the wing player. Further, we make the reasonable assumption that the risk of a counter attack from a missed back court attempt is the same as that of a missed wing attempt. As a consequence, the potential is equal to the shooting efficiency minus the same constant for both back court and wing players. By the Tactical Theorem of Team Sports, the optimal proportion of back court player shots is given at the point where the potential frequency functions satisfy *f*_*b*_(*x*_*b*_) = *f*_*w*_(1 − *x*_*b*_). In general, this suggests that the players with high efficiency should most likely be the ones to pursue additional shot opportunities, at the expense of the players with lower efficiency. Hence, the more efficient players should be put in shot positions more often, if that is at all possible without dramatically decreasing their efficiency to levels below those of the less efficient players. The opposite is true for less efficient players, who should take fewer shots and focus on getting their efficiency up. For a team in optimal play, all positions feature equal potential for their best shot opportunity not pursued.

It is natural to assume that the potential frequency functions are convex, meaning in essence that it is always easier to find bad shot opportunities than good ones. Under this assumption, the efficiency for all players will be quite close to the potential frequency function threshold at which to cease taking shots. To illustrate this using the setting of the present example, the efficiency for the back court and wing players will be larger than, but close to, *f*_*b*_(*x*_*b*_). But this implies that the “truths” stated in the beginning of the example are not truths at all, but rather signs of a non-optimal team strategy.

The main argument for accepting less efficiency for back court shots is that the defense will start to move up court if expecting additional shots from those positions. This results in more space for the efficient pivot players. However, by the Positioning for Indifference lemma, an optimal defense will only re-position for offensive alternatives that have a potential equal to the relevant alternatives. Hence, shots that are fired from back court which feature a potential below some efficiency threshold should not cause the defense to adjust their positions, but rather be appreciated by the defense as a non-optimal offensive strategy.

#### Team handball; wing change over

**Fig 2:**
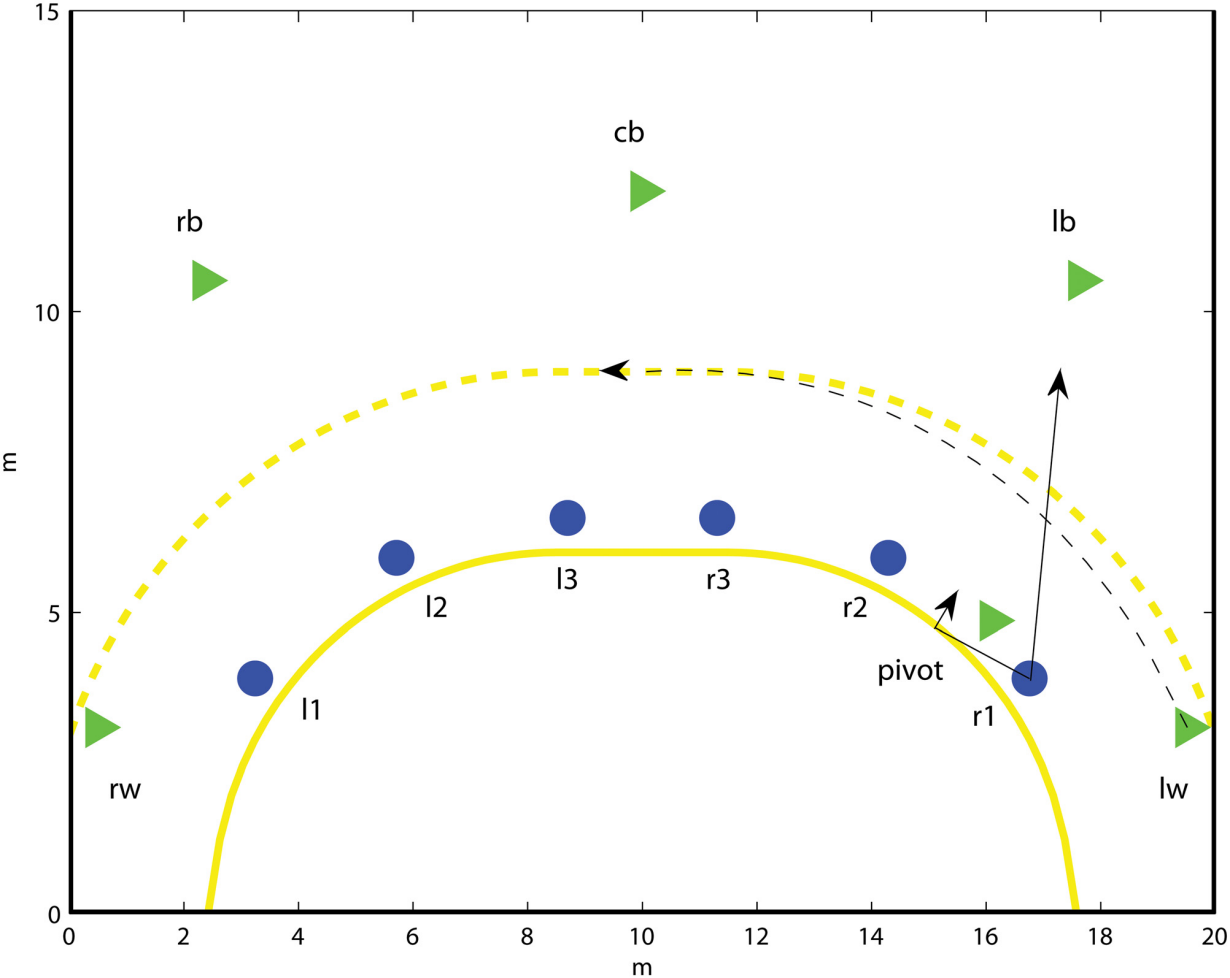
Schematic figure of a wing changeover opening in team handball. The offensive players are denoted by triangles and the defensive players are marked by cirles. The dashed line displays the movement path of lw and the solid lines refer to the possible moves for r1.

Consider a wing change over, which is a frequently occurring offensive opening in team handball. In a wing change over, one of team A’s wings repositions, with or without ball possession, to the opposite side of the court. The pivot subsequently screens on the inner side of r1, see Fig 2. We denote the players contributing in offensive play by: left wing (lw), left side back (lb), center back (cb), right side back (rb), right wing (rw) and pivot. The defensive positions are numbered from the side: left 1 (l1), left 2 (l2), left 3 (l3), right 3 (r3), right 2 (r2), right 1 (r1).

The r1 defender chooses either to advance up court, marking the lb player, or to battle the pivot in an attempt to get around the screening and remove the goalscoring threat from the screening pivot. In the first case, r2 is responsible for handling the pivot, and these two players are usually of comparable size and strengths. However, in the latter case, it is the role of r1 to take care of the pivot. This is typically a mismatch situation where the big pivot player has a physical advantage over r1. Given r1 stays flat, team A will launch the attack from the lb player. If r1 moves up court, team A decides between launching the attack from the marked lb player, or from a central position. We can model this as follows. We denote by *v*_*p*_ the potential of the situation where the pivot is screening r1. The potential of an attack started from the lb is *v*_*b*_(*y*), where *y* is the position of r1 as the situation begins. Further, *v*_*c*_(*y*) is the potential of the situation where the attack is started from a central position, without lb. The potential, *v*_*b*_(*y*), decreases as r1 approaches lb. However, the farther away r1 is from the remaining part of the defense, the more sparsely they will have to position themselves to cover the whole 6m line. Hence, in this case the potential *v*_*c*_ increases.

**Fig 3:**
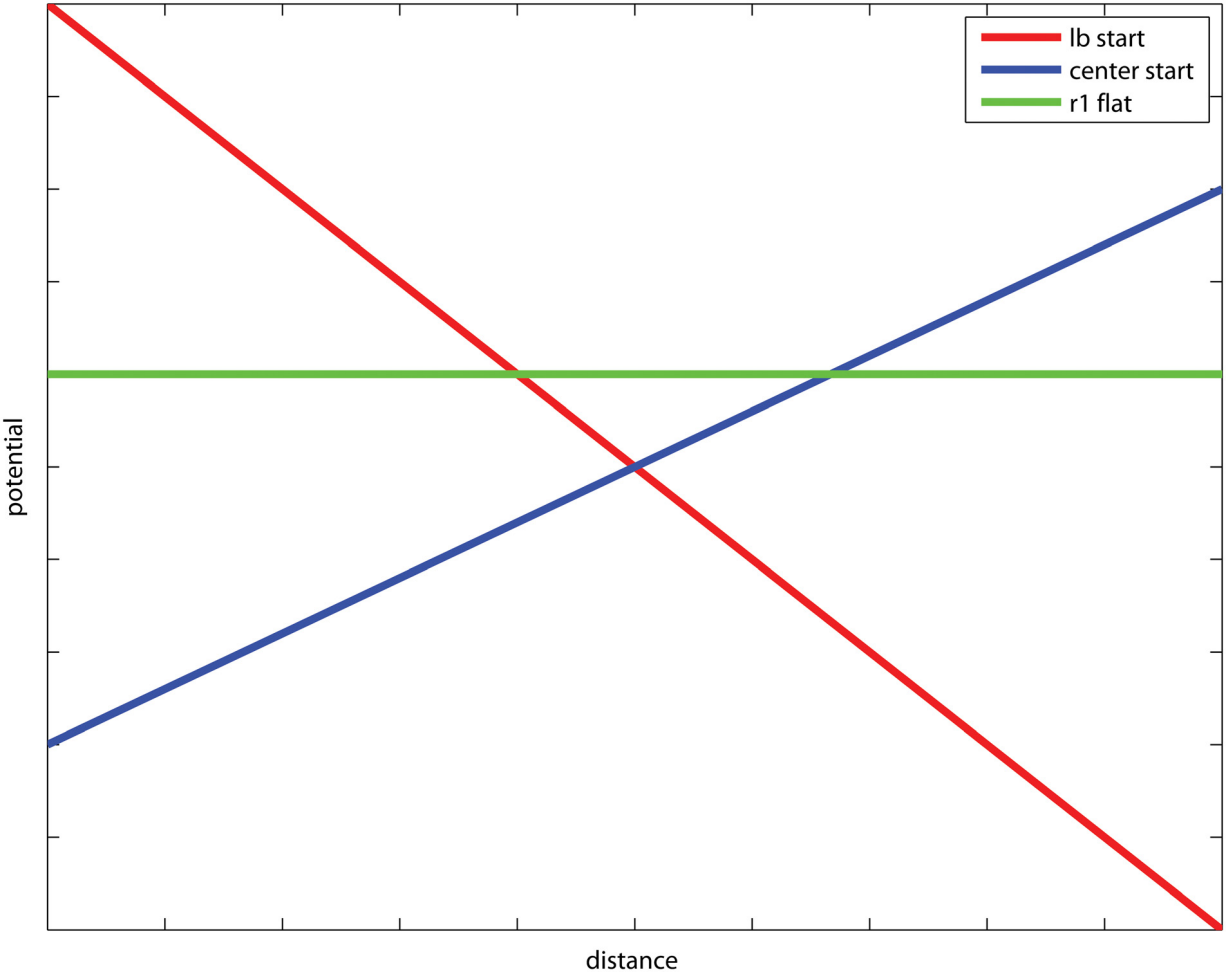
The potential functions for the example *Team handball; wing change over*. The potential for a lb start (red) and a center start (blue), respectively, given that r1 moves up court. For reference, the potential of the situation where r1 stays flat (green) is displayed, even though it is not a function of *y*.

In order to illustrate the optimality principle, we let the potential functions be linear in the distance that r1 lifts from the field line, where *v*_*b*_(*y*) decreases and *v*_*c*_(*y*) increases with respect to the how far up court r1 moves, see Fig 3.

Given that r1 lifts up court and none of offensive alternatives are dominating, we have by the Positioning for indifference lemma that the potential of the situation is minimized for y* such that *v*_*b*_(y*) = *v*_*c*_(y*). Further, r1 should always lift up court if *v*_*b*_(γ*) < *v*_*p*_, and stay flat otherwise.

In reality, dichotomous choices to be flat or move up court are typically set at tactics sessions prior to the game. Here, team tactics are often that the speedy wing defenders should seek to avoid the screening situation associated with staying flat by choosing the alternative to move up court. Further, the offensive side attempts to hide the opening, sending over the offensive wing player lw to its right simultaneously as the lb starts the attack. This has the effect that r1 may not able to reach the optimal position prior to when lb charges. Further, r1 needs to re-categorize the game situation; to decide to move up court but only to a limited stretch $\tilde{y}<y^{*}$, or to stay flat and challenge the screening set by the pivot. In the particular game situation displayed in Fig 3, then the optimal defensive strategy is to stay flat if $\tilde{y}$ yields a potential $v_{b}\left(\tilde{y}\right)<v_{p}$.

Note that the vast majority of wing defenders who choose to advance up court, do this all the way up to the side back court players, taking them out completely. This will only be the optimal defensive strategy given that the corresponding side back court player is the dominating alternative.

#### Ice hockey; one against one

In ice hockey, it is a common situation that a single offensive player with possession faces a single defender. Here, we will draw conclusions from the Positioning for Indifference lemma in a simplified such game situation. The offensive player, *A*_1_, may choose to either shoot or to attempt to dribble past the defender, *B*_1_. We assume that regardless of how possession is lost, either by a missed shot or a failed dribble, the resulting potential will be the same. This is a natural assumption, since in either case there are four players in team A defending the counter attack. An attempt to dribble has a certain probability of failure, resulting in a loss of possession. However, if it succeeds, *A*_1_ has a free path up to the goal. This is a situation with a very high probability of scoring, and thus also high potential.

The defending *B*_1_ player has to choose how to position herself in stopping each of the two offensive alternatives. If *B*_1_ puts much pressure on *A*_1_ early on, by seeking to close the distance between the players, the offensive choice of shooting will be bad, since *A*_1_ is far away from the goal and will have to take a shot under pressure. On the other hand, when *B*_1_ puts high pressure on *A*_1_, the relative speed between the players will be large. This improves the probability of a successful dribble, since *B*_1_ will have less time to intercept the puck before *A*_1_ has passed by. Further, there is much space between the players and the goal for *A*_1_ to use if she chooses this option. Hence, the dribbling alternative will be better the more pressure *B*_1_ puts on *A*_1_. In addition, if *B*_1_ chooses to fall back and maintain a low relative speed versus *A*_1_, the option to dribble will become worse. Simultaneously, the shot alternative will become better since *A*_1_ can come closer to the goal undisturbed.

Given that neither of the two offensive alternatives are dominating, by the Positioning for Indifference lemma *B*_1_ should put pressure on *A*_1_ to the extent that the two options, to shoot or to dribble, yield the same potential. This is analogous to the red and blue lines in Fig 3. We call this position y*. By playing y*, *B*_1_ has minimized the potential of the best choice for *A*_1_, and hence plays optimally. Note that *A*_1_ might still score regardless of what *B*_1_ does. However, playing similar situations many times, following the effort y* will result in the least number of goals scored by team A.

## Strategic game situations

In this section, we will set up a strategic game theoretic framework to model various game situations. Again, there are two teams in the game, team A and team B.

To conduct our analysis we need some definitions and results from game theory, which we present below. These are completely standard, see e.g. [18]. We are considering strategic zero-sum game situations, which are of a one-time choice type. Zero-sum games are games for which the utilities of one side is exactly the negative of that of the other side. We will define the team A utility function to be the potential, and hence the team B utility function is the negative potential.

A strategic game situation is a situation where team A and team B each have a choice to make and none of the participators know the opponent’s actions in advance. Hence, the participators make decisions simultaneously and independently, and none of the players have prior information of their counterpart’s choices.

**Definition 6 (Strategic zero sum game)** *A strategic zero sum game is a structure,* $\langle \Pi ,\Gamma ,\tilde{v}\rangle$, *consisting of the following components:*

- *two sets of actions,* Π *and* Γ, *that team A and team B choose from, respectively.*
- *a potential function* $\tilde{v}:\Pi \times \Gamma \to [-1,1]$

Further, we associate to a strategic game a set of team A *strategies*, *π* ∈ 𝓛(Π), on the actions in Π, i.e. a team A strategy for a strategic game is a probability distribution on the set of offensive actions. For a finite set Π, then *π* = (*π*_1_, *π*_2_, …, *π*_*n*_), *π*_*i*_ ≥ 0 and ∑_*i*_ *π*_*i*_ = 1 for the *n* actions in Π. Analogously, team B has a set of defensive strategies, *γ* ∈ 𝓛(Γ). The potential associated with the strategies (*π*, *γ*) are

$$\begin{array}{c} v\left(\pi ,\gamma \right):=𝔼[\tilde{v}\left(X,Y\right)], \end{array}$$

where *X* has distribution *π* ∈ 𝓛(Π), and *Y* has distribution *γ* ∈ 𝓛(Γ). The notation *v* defines the potential of the game for the pair of strategies (*π*, *γ*).

We will have use of the following definitions.

**Definition 7 (Nash equilibrium)** For a game situation of strategic type, a set of strategies (π*, γ*) for team A and team B, respectively, is called a **Nash equilibrium** if neither side achieves a higher potential by single-handedly deviating from the strategy, i.e.if

$$\begin{array}{ccc} v(\pi^{*},\gamma^{*}) & \leq & v(\pi^{*},\gamma )\\ v(\pi^{*},\gamma^{*}) & \geq & v(\pi ,\gamma^{*}), \end{array}$$ (4)

*for* *π* ∈ Π *and* *γ* ∈ Γ.

**Definition 8 (max-min strategy)** *A team A **max-min strategy** for a strategic game* $\langle \Pi ,\Gamma ,\tilde{v}\rangle$ *is the strategy* π* ∈ Π *that maximizes the function*

$$\begin{array}{c} f\left(x\right)=\min_{\gamma \in 𝓛(\Gamma )}v\left(x,\gamma \right). \end{array}$$

The value f(π*) is called the team A safety level. Analogously, a team B max-min strategy for the same game is the strategy γ* ∈ Γ *that maximizes*

$$\begin{array}{c} g\left(y\right)=\min_{\pi \in 𝓛(\Pi )}\{-v(\pi ,y)\}, \end{array}$$

*i e, it maximizes the negative of the potential. Hence,*

$$\begin{array}{c} g(\gamma^{*})=-\min_{\gamma \in 𝓛(\Gamma )}\max_{\pi \in 𝓛(\Pi )}v\left(\pi ,y\right), \end{array}$$

so we call γ* a **min-max strategy**. The value g(γ*) is called the team B safety level.

It can be shown that, at a Nash equilibrium, the team A safety level coincide with the negative of the team B safety level. We get this and more by the following theorems.

**Theorem 9 (Max-Min Theorem)**

- *For each game situation* $\langle \Pi ,\Gamma ,\tilde{v}\rangle$, *there exists a Nash equilibrium.*
- A strategy vector (π*, γ*) in a game situation $\langle \Pi ,\Gamma ,\tilde{v}\rangle$ is a Nash equilibrium if and only if π* is a max-min-strategy for team A and γ* is a min-max-strategy for team B.
- *The potential in a Nash equilibrium is equal to the safety level of team A,* *f*(π*), and *f*(π*) = −*g*(γ*).

The Max-Min theorem gives that the concept of Nash equilibria is a stable and satisfying solution to zero-sum games, since both teams can decide their optimal strategy without considering the strategy of the other team.

The following theorem is a strategic game analogue of the Positioning for Indifference lemma.

**Theorem 10 (Indifference Principle)** A game situation with a Nash equilibrium (π*, γ*) and potential $\tilde{v}$ has safety level v* if and only if

$$\begin{array}{ccc} \pi_{i}^{*}>0 & \Rightarrow & \sum_{j\in \Gamma }\tilde{v}\left(i,j\right)\gamma_{j}^{*}=v^{*}\\ \pi_{i}^{*}=0 & \Rightarrow & \sum_{j\in \Gamma }\tilde{v}\left(i,j\right)\gamma_{j}^{*}\leq v^{*}\\ \gamma_{j}^{*}>0 & \Rightarrow & \sum_{i\in \Pi }\tilde{v}\left(i,j\right)\pi_{i}^{*}=v^{*}\\ \gamma_{j}^{*}=0 & \Rightarrow & \sum_{i\in \Pi }\tilde{v}\left(i,j\right)\pi_{i}^{*}\geq v^{*}. \end{array}$$

**Definition 11 (deterministic action)** *An action a* ∈ 𝓛(Π) for which *a*(*i*) = 1 *for some* *i* = 1, …, *n* *is called a **deterministic action**.*

**Definition 12 (strictly dominated action)** *A team A deterministic action* *a* ∈ 𝓛(Π) *such that v(a, γ) < v(π>, γ) for all team B strategies γ and some team A strategy π, is called a **strictly dominated action**. Conversely, a deterministic action for team B,* *d* ∈ 𝓛(Γ), *is strictly dominated if v(π, d) > v(π, γ) for all team A strategies π and some team B strategy γ*.

A consequence of the Indifference Principle is that strictly dominated actions are assigned probability 0 in a Nash equilibrium.

We have now laid out the theoretic framework for the strategic games in our setting.

### The Indifference Principle applied to strategic games

We summarize now the results of the previous section in a theorem. The theorem follows directly from our model setting and standard results of game theory. It states that for a given game situation, with utility given by the potential, the optimal strategy for both the offense and the defense is a Nash equilibrium.

**Theorem 13 (Fundamental Principle of Strategic Games in Team Sports)** *For a strategic game situation* $\langle \Pi ,\Gamma ,\tilde{v}\rangle$ *with team A strategy π* ∈ 𝓛(Π), *and team B strategy γ* ∈ 𝓛(Γ), *the **potential** is the probability of team A scoring next goal minus the probability of team B scoring next goal*,

$$\begin{array}{c} v(\pi ,\gamma )=𝔼[V(T)|\pi ,\gamma ]. \end{array}$$

The Nash equilibrium strategy π* *for team A is the strategy that maximizes the minimal potential*,

$$\begin{array}{c} \pi^{*}=\arg\max_{\pi \in 𝓛(\Pi )}\min_{\gamma \in 𝓛(\Gamma )}v\left(\pi ,\gamma \right). \end{array}$$

Further, the Nash equilibrium strategy γ* *for team B minimizes the maximum potential such that*

$$\begin{array}{c} \gamma^{*}=\arg\min_{\gamma \in 𝓛(\Gamma )}\max_{\pi \in 𝓛(\Pi )}v\left(\pi ,\gamma \right). \end{array}$$

**Proof**. The result follows immediately from the Max-Min theorem.■

The strategy γ* guarantees team B the highest possible potential it can obtain without knowledge of the team A strategy. By the Max-Min theorem, the Nash equilibrium is given by the minimax strategy. Hence, analogously to the Positioning for Indifference lemma, the optimal defensive strategy is to make the best alternative for the offense as bad as possible.

### Notable examples

In this section we aim to convey the power of applying the Fundamental Principle of Strategic Games in Team Sports to various game situations in ice hockey.

Here we will break down game situations to categorical strategic games. Recall that a strategic game is a choice situation where the participators make their decisions simultaneously and have no prior information of the opponents actions.

#### Ice hockey; chasing the puck

A frequently occurring situation in ice hockey is that a team A player shoots the puck into her offensive corner, and a player from each team, *A*_1_ and *B*_1_, chases after it. At some point, the two players are faced with a choice to either charge forward in an attempt to win the puck or to hold back and by doing so invite the opposing player to go first into the situation. This can be modeled as a strategic game defined by

$$\begin{array}{c} G^{c}=\langle \left\{charge,hold\;back\right\},\left\{charge,hold\;back\right\},\tilde{v}\rangle , \end{array}$$

where $\tilde{v}$ is the potential defined on each pair of offensive and defensive actions. We denote by *v*_*A*_ the potential of the situation where *A*_1_ wins the puck when both players charge. Further, *v*_*B*_ is the potential when *B*_1_ wins the puck, regardless of the manner by which this happens. Finally, *v*_*h*_ is the potential should *A*_1_ charge and *B*_1_ hold back. In this case, *B*_1_ avoids to tackle in the first instance, and instead positions herself, in balance, within stick reaching distance to *A*_1_ and the puck. Obviously, *v*_*B*_ < *v*_*h*_ < *v*_*A*_, since it is better for team A to have puck possession than to not have it, and since *A*_1_ is in a better position to score if *B*_1_ is not between *A*_1_ and the goal. Additionally, *p* is the probability for *A*_1_ to win the situation given that both players charge and *q* is the probability for *A*_1_ to win possession given that both players hold back. The game is illustrated by the matrix given in Table 1.

**Table 1:** The matrix of the strategic game *G* ^*c*^.

	team B		
		charge	hold back
team A	charge	*pv*_*A*_ + (1 − *p*)*v*_*B*_	*v*_*h*_
	hold back	*v*_*B*_	*qv*_*h*_ + (1 − *q*)*v*_*B*_

Since the *A*_1_ action to hold back is strictly dominated by the charge alternative, the Indifference Principle yields that the Nash equilibrium contains only the charge action for *A*_1_. Given that

$$\begin{array}{c} v_{h}<pv_{A}+\left(1-p\right)v_{B} \end{array}$$

then there is a Nash equilibrium at π* = (1, 0), γ* = (0, 1) and the optimal strategy for *A*_1_ is to charge while the optimal *B*_1_ strategy is to hold back. Conversely, given that

$$\begin{array}{c} v_{h}>pv_{A}+\left(1-p\right)v_{B}, \end{array}$$

there is a Nash equilibrium at π* = (1, 0), γ* = (1, 0) so it is optimal for both players to charge in the situation.

In this game situation, intuition leads us to believe that

$$\begin{array}{c} v_{B}\leq v_{h}<<v_{A}, \end{array}$$

since the resulting scoring chance if the offensive player has possession with the defender out of the way is much higher than for any of the alternative outcomes. Further, if both players were to choose to charge, they weigh approximately the same, and they come into the situation with the same speed, then it seems to be approximately a 0.5 probability for *B*_1_ to win the puck possession. Hence, *B*_1_ should hold back in all such situations.

#### Ice hockey; pass or dribble

An important choice that all players need to make in many team sports, is to decide when to pass and when to dribble. We consider this problem for ice hockey in a strategic game setting. However, the analysis is valid for other sports as well, e g soccer, team handball, and basketball. Consider a situation where team A has puck possession. The team A player *A*_1_ with the puck has two choices, to pass or to dribble. Team B on the other hand can decide between to put pressure on *A*_1_, or to hold back and wait until team A comes closer to the team B goal. This typically allows team B to defend themselves in a more compact and efficient manner, at the expense of that team A can advance forward. The game is defined by

$$\begin{array}{c} G^{p}=\langle \left\{pass,dribble\right\},\left\{press,hold\;back\right\},\tilde{v}\rangle , \end{array}$$

where $\tilde{v}$ is the potential defined on each pair of offensive and defensive actions. The matrix for the game is given in Table 2.

**Table 2:** The matrix of the strategic game *G* ^*p*^.

	team B		
		press	hold back
team A	pass	*pv*_*Ap*_ + (1 − *p*)*v*_*Bp*_	*v*_*ph*_
	dribble	*qv*_*Ad*_ + (1 − *q*)*v*_*Bd*_	*v*_*dh*_

Now, we assume the typical situation that *A*_1_ has sought protection behind her own goal, and is now advancing with the rest of team A ahead of her. We assume further that *v*_*ph*_ and *v*_*dh*_ are such that it is optimal for team B to start to put pressure on *A*_1_. In addition, we assume that *p* > *q*, which is natural since it is most often considerably easier to succeed with a pass than with a dribble. Finally, we set *v*_*Bp*_ > *v*_*Bd*_. This implies that the situation resulting from an intercepted pass, *v*_*Bp*_, is better for team A than if *A*_1_ is to fail with a dribble, *v*_*Bd*_. Obviously, we have also that *v*_*Ap*_ > *v*_*Bp*_ and that *v*_*Ad*_ > *v*_*Bd*_. Given the present situation, *v*_*Ap*_ ≈ *v*_*Ad*_, since not much is won from a successful dribble early on in an attack. If anything, one might suspect that *v*_*Ap*_ ≥ *v*_*Ad*_, as it takes a few seconds to complete a dribble, during which team B has the time to adjust its positions and put increasing pressure on the rest of team A. This implies that it would never be optimal to dribble, since *pv*_*Ap*_ + (1 − *p*)*v*_*Bp*_ > *qv*_*Ad*_ + (1 − *q*)*v*_*Bd*_. In fact, we need *v*_*Ap*_ < < *v*_*Ad*_ if the decision to dribble is going to be the best choice. Note also that this simple analysis suggests that given that team B puts pressure on *A*_1_, she maximizes her potential by maximizing the probability of a successful pass. Since a successful pass depends not only on the passer, but also on the receiver and the opponents, the only way to reach a high pass success rate *p* is to pass early. The reason is of course that team B will then have little chance to intercept the pass or to force *A*_1_ to dribble.

## Extensive game situations

More elaborate sport situations, where the offense and defense make sequential choices, can be modeled with so-called extensive games. We need to introduce some additional game theoretic notation for this setting. Again, these are standard, see e g [18].

**Definition 14 (Game tree)** *A game tree 〈P, p_0_, f〉 is a structure consisting of the following components:*

- *a non-empty set P, the elements of P are called positions;*
- *an element p_0_ ∈ P, the game starting position;*
- *a function f:P → 𝓟(P) from the set of positions to the power set of P i.e. all subsets of P.*

*The positions in f(p) are called **direct followers** to p. Furthermore, f has the property that for each position p ≠ p_0_ there is a unique sequence* ${\left(p_{k}\right)}_{k=0}^{n}$ *with p_0_ the starting position and p_n_ = p and p_k+1_ is a direct follower to p_k_ for k = 0, 1, …, n* − 1. *Any position q which can be reached from p is called a **follower** to p. We denote the pair (p, q) consisting of a position and any of its direct followers a **move**. The set of ending positions, i.e. positions with no followers, is denoted P_e_ and positions in P_i_ = P\P_e_ are denoted inner positions.*

**Definition 15 (Extensive game)** *An extensive game* $\langle T,t,{\left\{\mu_{p}\right\}}_{p:t(p)=c},\tilde{v}\rangle$ *is a structure consisting of the following components:*

- *a game tree T = 〈P, p_0_, f〉,*
- *a function t:P_i_ → {a, b, c} which determines the turn-order; whether the choice in the position is to be made is by the team A, team B or a random outcome.*
- *probability distributions μ_p_ over the direct followers of p for all positions such that t(p) = c.*
- *the potential,* $\tilde{v}$, *in the ending positions of the game tree.*

**Fig 4:**
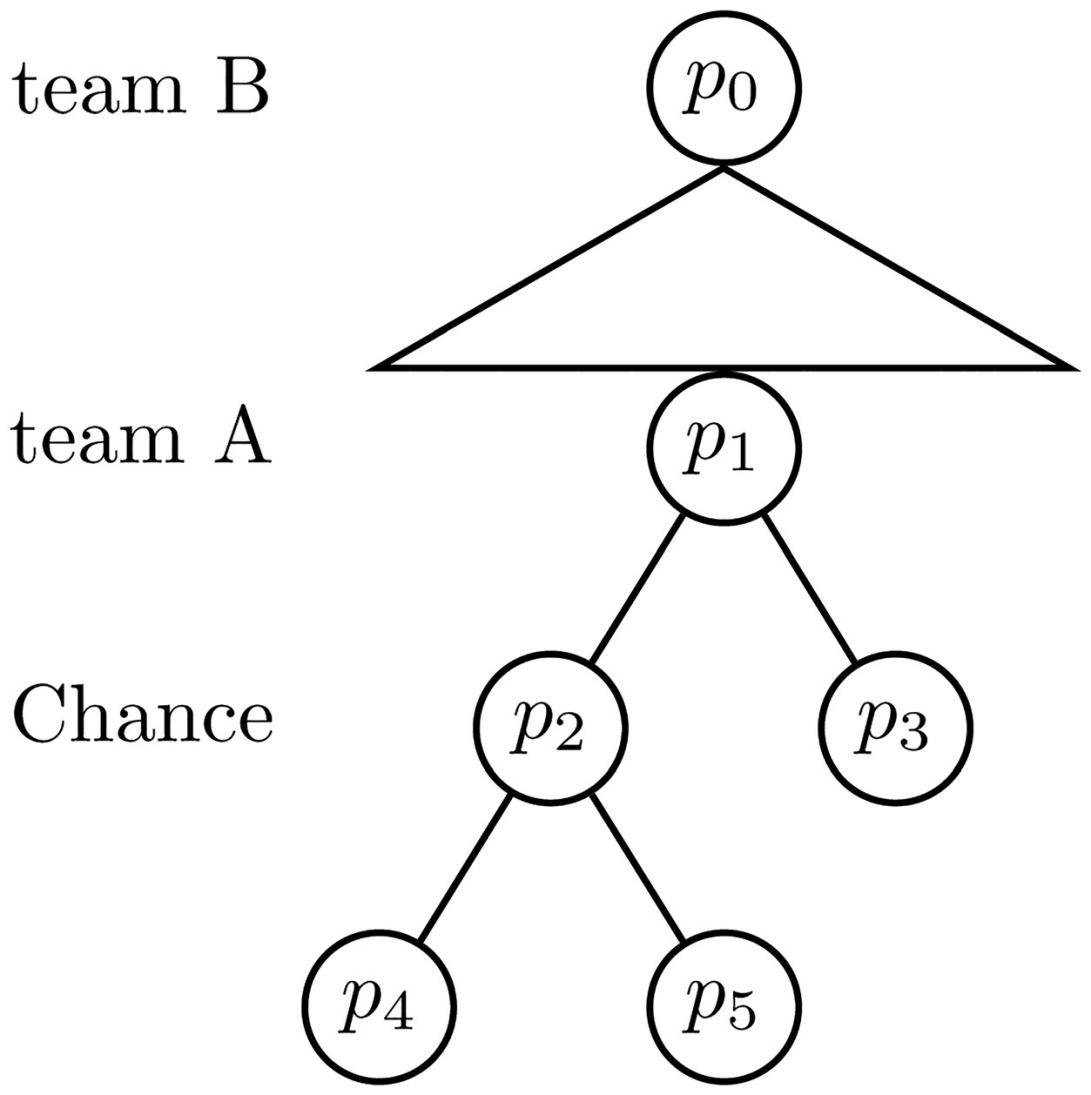
A game tree. An extensive game where *p*_0_ is the starting position, and *p*_0_ (team B), *p*_1_ (team A) and *p*_2_ (chance) are inner positions. Here, team B has a decision in continuum while the team A and chance moves are categorical.

Extensive games may be illustrated in game trees where the turn order is displayed. Here, direct followers are displayed by connections between the nodes, and triangles denote actions in continuum (such as choice of position or velocity). Further, lines denote categorical actions, such as to shoot or to pass, see Fig 4.

**Definition 16 (Subgame)** *For p ∈ P, the restriction of a game G starting in p is called a subgame G_p_ to G*.

**Definition 17 (Team positions)** *The positions p ∈ P_i_ such that t(p) = a are called **team A positions** and denoted Π, analogously are Γ = {p ∈ P_i_:t(p) = b} called **team B positions**, and Λ = {p ∈ P_i_:t(p) = c} called **chance positions**.*

**Definition 18 (Strategy)** *A **team A strategy**,* *σ*_*A*_:Π ↦ 𝓛(*P*) *in a game situation on extensive form is a function with the property that σ_A_(p) assigns probabilities to all direct followers q ∈ f(p), for every team A position p. I.e. σ_A_ gives the probabilistic decision path for team A, for all its possible positions. Analogously, a **team B strategy**, σ_B_, defines the corresponding probabilistic decision path of team B.*

We will denote by 𝓛(Π) and 𝓛(Γ) the set of all team A and team B strategies, respectively. The potential of the game, given strategies *π* and *γ*, is set to be

$$\begin{array}{c} v\left(\pi ,\gamma \right)=𝔼[\tilde{v}\left(X,Y\right)], \end{array}$$

where *X* has distribution *π* ∈ 𝓛(Π) and *Y* has distribution *γ* ∈ 𝓛(Γ).

**Definition 19 (Nash equilibrium for extensive game situation)** *For an extensive game* $\langle T,t,{\left\{\mu_{p}\right\}}_{p:t(p)=c},\tilde{v}\rangle$, strategy vectors (π*, γ*) such that π* = 𝓛(Π) *and* γ* = 𝓛(Γ) *are called a Nash equilibrium if*

$$\begin{array}{c} v\left(\pi ,\gamma^{*}\right)\leq v\left(\pi^{*},\gamma^{*}\right)\leq v\left(\pi^{*},\gamma \right), \end{array}$$

*for all strategies* *π* = 𝓛(Π) *and* *γ* = 𝓛(Γ). Further, if the restriction of π*, γ* to every subgame is a Nash equilibrium in that game, it is called a **subgame perfect Nash equilibrium**.

### The Indifference Principle applied to extensive games

We summarize here the results of the previous section in a theorem, which is an immediate consequence of our game theoretic model. The theorem states that for a given game situation, with utility given by the potential, the optimal strategy for both the offense and the defense is a subgame perfect Nash equilibrium.

**Theorem 20 (Fundamental Principle of Extensive Games in Team Sports)** *For an extensive game situation* $G=\langle T,t,{\left\{\mu_{p}\right\}}_{p:t(p)=c},\tilde{v}\rangle$ the subgame perfect Nash equilibrium strategy π* *for team A is the strategy that maximizes the minimal potential*,

$$\begin{array}{c} \pi^{*}=\arg\max_{\pi \in 𝓛(\Pi )}\min_{\gamma \in 𝓛(\Gamma )}v\left(\pi ,\gamma \right), \end{array}$$

for every subgame G_p_. Further, the subgame perfect Nash equilibrium strategy γ* for team B minimizes the maximal potential such that

$$\begin{array}{c} \gamma^{*}=\arg\min_{\gamma \in 𝓛(\Gamma )}\max_{\pi \in 𝓛(\Pi )}v\left(\pi ,\gamma \right), \end{array}$$

*for every subgame G_p_.*

**Proof**. The result follows immediately from the definition of subgame perfect Nash equilibrium.■

Completely analogously to the strategic game setting, the strategy γ* guarantees that team B gets the lowest possible potential it can achieve without knowing anything about the team A strategy. Hence, the result above extends the Positioning for Indifference lemma such that for sequential moves, the optimal defensive strategy is to make the best alternative for the offense as bad as possible.

### Notable examples

We analyze now a few examples where each team can make sequential choices.

#### Ice hockey; two against one

**Fig 5:**
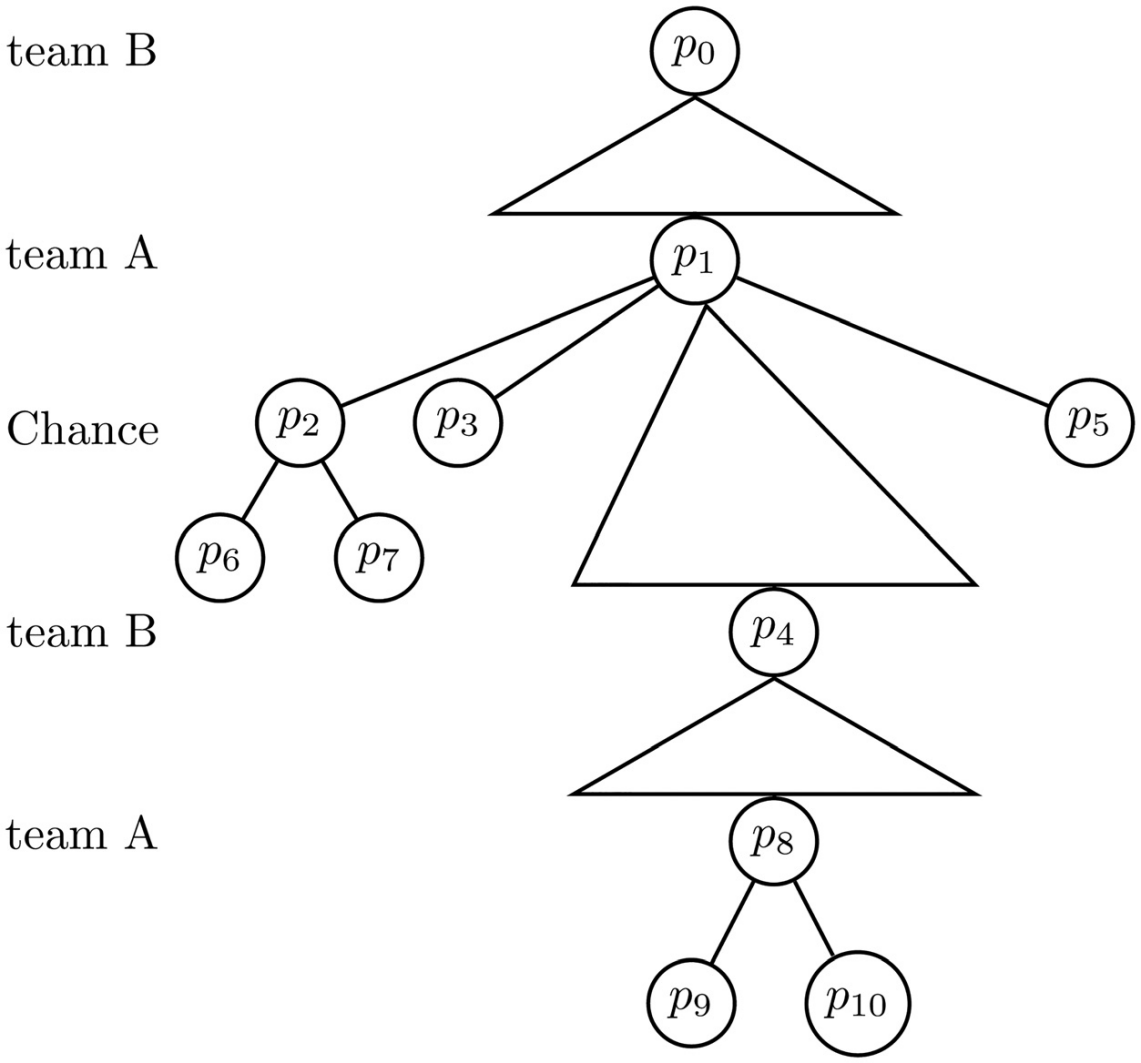
The extensive game tree in the example *Ice hockey; two against one*.

In ice hockey, the game situation where two team A players *A*_1_ and *A*_2_ face a single team B defender *B*_1_ occurs often. We assume that *A*_1_ has initial puck possession. Due to the blue line offside, the situation starts as a one against one situation where *B*_1_ positions herself with the move (*p*_0_, *p*_1_) to prevent the attack. Next, *A*_1_ can choose either of the following four moves: to dribble (*p*_1_, *p*_2_); to shoot (*p*_1_, *p*_3_); to avoid *B*_1_, (*p*_1_, *p*_4_); and to pass *A*_2_, (*p*_1_, *p*_5_). If *A*_1_ decides to dribble then she is either successful, (*p*_2_, *p*_6_), or looses possession, (*p*_2_, *p*_7_). Further, if *A*_1_ shoots, she ends up in *p*_3_, an ending position. If *A*_1_ makes the move to avoid *B*_1_, (*p*_1_, *p*_4_), then *B*_1_ re-positions with the move (*p*_4_, *p*_8_). The subgame situation is ended by a shot from *A*_1_, (*p*_8_, *p*_9_), or with a pass to *A*_2_ for her to shoot a one timer, (*p*_8_, *p*_10_). Finally, if *A*_1_ passes *A*_2_, (*p*_1_, *p*_5_), we are in a new two against one situation, but where *A*_2_ has puck possession. The corresponding game is illustrated in Fig 5, where a description of the positions are given in Table 3.

**Table 3:** Description of positions for the example *Ice hockey; two against one*.

Positions	Description
*p*_0_	team B position, chooses a position in continuum.
*p*_1_	team A position, chooses categorically to dribble, to shoot, to pass, or to take a new position to avoid confrontation.
*p*_2_	chance position, dribble move.
*p*_3_	ending position, shot by *A*_1_.
*p*_4_	team B position, chooses a position in continuum.
*p*_5_	pass to *A*_2_, the game starts over in *p*_0_ with new positions.
*p*_6_	ending position, successful dribble.
*p*_7_	ending position, failed dribble.
*p*_8_	team A position, chooses categorically to shot or to pass.
*p*_9_	ending position, shot by *A*_1_.
*p*_10_	ending position, shot by *A*_2_.

Note that there is a certain amount of subjectivity regarding how many nodes to include, and which ones.

**Fig 6:**
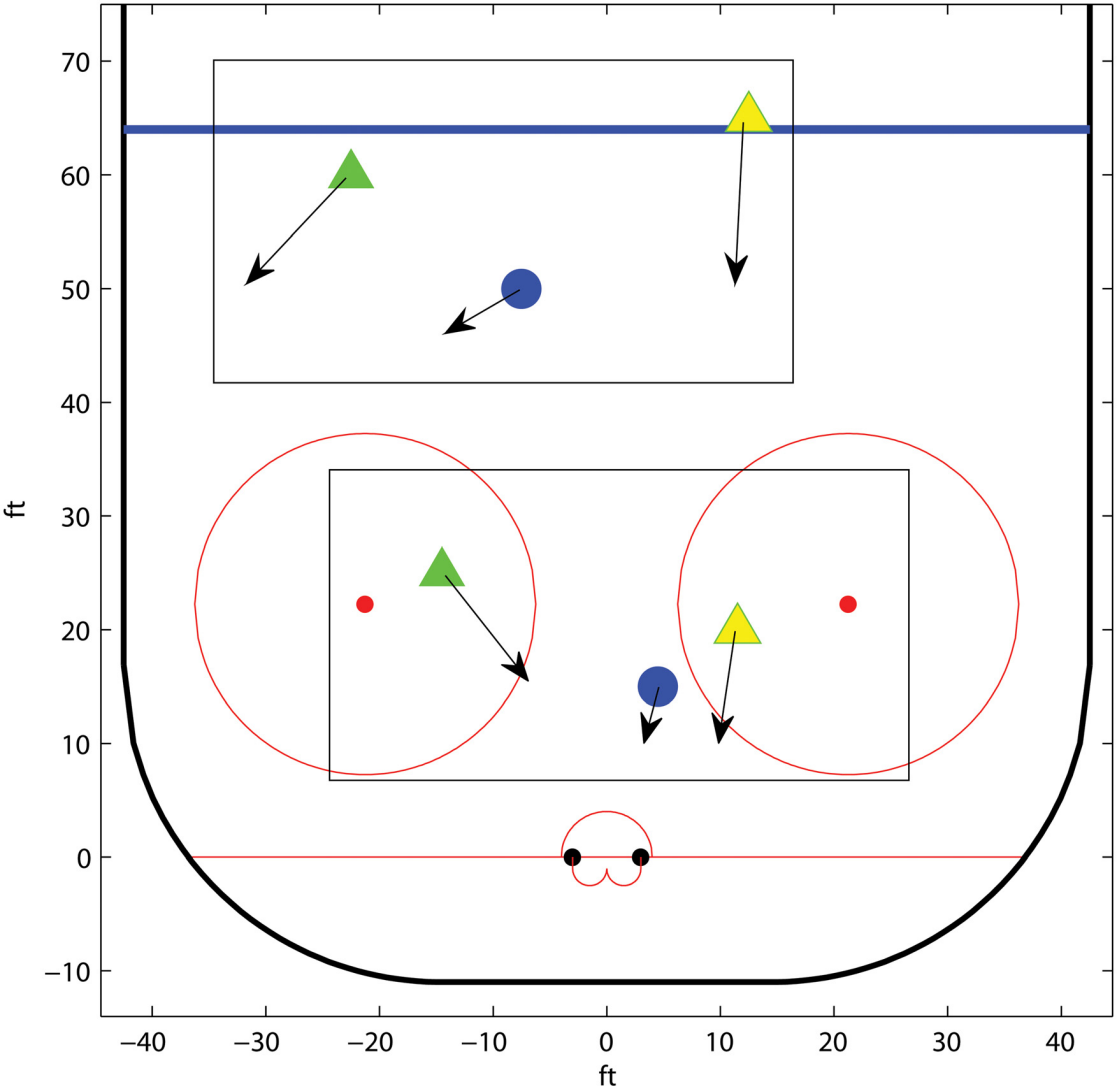
Outline of the optimal trajectories in the example *Ice hockey; two against one*. The offensive players are denoted by triangles, where a solid triangle marks the possession holder, and the defender is denoted by a circle. In the upper rectangle, *B*_1_ puts pressure on the puck holder *A*_1_, who avoids confrontation to await *A*_2_. In the lower rectangle, having steared *A*_1_ into a worse position, *B*_1_ shifts focus to *A*_2_, in order to decrease the greater threat posed by that player.

As in the one against one example, we assume that regardless of how possession is lost, the resulting potential will be the same. Note that if the potential of the subgames starting in positions *p*_4_ and *p*_5_ are dominated by the subgames started in the alternatives *p*_4_ and *p*_5_, the present game will be played identically to the one against one example. However, in reality the opposite is true; *A*_1_ knows that team A benefits considerably from being two against one, rather than one against one, so a rational *A*_1_ will avoid the moves (*p*_1_, *p*_2_) and (*p*_1_, *p*_3_) in the first instance. The player *B*_1_ knows this, too, and can therefore put more pressure on *A*_1_ in order to force *A*_1_ towards the side of the rink, to a position with smaller potential. The defender *B*_1_ can do this to the extent that a pass back to *A*_2_ will yield an equally good potential. Hence, to pass and to avoid are the relevant alternatives for *A*_1_ in node *p*_1_. The potential of the ending situation is strongly dependent on the position of *A*_1_ in node *p*_4_. The subgame started at *p*_4_ will have smaller potential the further out to the side *A*_1_ has been forced by *B*_1_. Next, due to the limited mobility of the goalkeeper, the position *p*_10_, a pass to *A*_2_, will have a very high potential. Conversely, the goalkeeper can save a large proportion of the shots coming from *A*_1_, due to that she has clear vision and can attain good positioning. Hence, since it is easier for *B*_1_ to intercept a pass if she is near *A*_2_, the optimal position for *B*_1_ will be close to *A*_2_, focusing primarily on preventing the pass from *A*_1_ to be completed. This is done to the extent that the potential of the two alternatives are equal, by the Positioning for Indifference lemma. Consequently, the defender *B*_1_ should always maintain focused on *A*_2_ until *A*_1_ is close enough to the goal so that *B*_1_ can put pressure on both *A*_1_ and *A*_2_ simultaneously. It follows that the optimal team B defender trajectory will be shaped like an S, see Fig 6.

The player *B*_1_ starts by putting aggressive pressure on *A*_1_—more so than if *A*_1_ was to come alone in a one against one in the same position—to force her to the side. She then withdraws to devote her main attention to prevent a pass to *A*_2_. Finally, *B*_1_ comes back to a position in front of her goal keeper. From here, *B*_1_ can both intercept a pass to *A*_2_ and stop *A*_1_ from advancing closer to the goal with the puck at the same time.

### Rationalizable beliefs

We indicate now a direction in which it is natural to expand the framework that we have developed so far.

We know that players deviating from the Nash equilibrium will invite the counterpart to improve the potential. For example, if team B knows that team A will follow a certain strategy $\hat{\pi }\in 𝓛(\Pi )$, then team B can lower the potential of the game situation by choosing the optimal defense $\text{arg}\;{\text{min}}_{\gamma \in 𝓛(\Gamma )}v(\hat{\pi },\gamma )$. Hence, it is distinctly good to have the ability to choose “late” in decision situations, which is a skill that professional players practice in many sports.

To illustrate further, consider the ice hockey example of one against one. If the defender knows that the offensive player is reluctant to dribble, then she can put more pressure early on, in order to stear the offensive player further away and to the sides. This renders that the potential of the game will be smaller than if team A played by the optimal Max-Min strategy.

As an example from team handball, we re-visit the “truth” that the wing players should have a much higher efficiency than the back court players. We argued earlier that this is likely to be a suboptimal strategy. If we assume that team A takes too few shots from some positions, then team B can focus more effort on making the remaining positions even worse, which has the effect that the players who take too large shot proportions will have trouble to maintain their shot efficiency.

## Shot potential

We introduce in this section the concept of a shot potential, which is related to the potential. The shot potential fields can be used to make numerical analysis of various game situations.

The present paper is to a large extent based on the concept of potential i.e. the probability of team A scoring next minus the probability of team B scoring next. However, in many situations the potential will be approximately equal to the probability of team A scoring, see e.g. the 2 against 1 example in Section 1. Thus the potential depends only on the probability of scoring, given the chosen strategies for each side. We make the following definitions.

**Definition 21 (Shot potential)** *The **shot potential** for A_1_ is the probability that A_1_ scores with an instant shot from her present position, given that the resulting situation if the shot is missed has potential 0.*

**Definition 22** *We refer to the level curves of the shot potential as **isolines***.

*Note that the isolines give sets of points for which the shot potential is equal.*

### Notable examples

Here we will use the shot potential to analyze several situations in ice hockey in a dynamic setting.

#### Ice hockey; eccentric isolines

**Fig 7:**
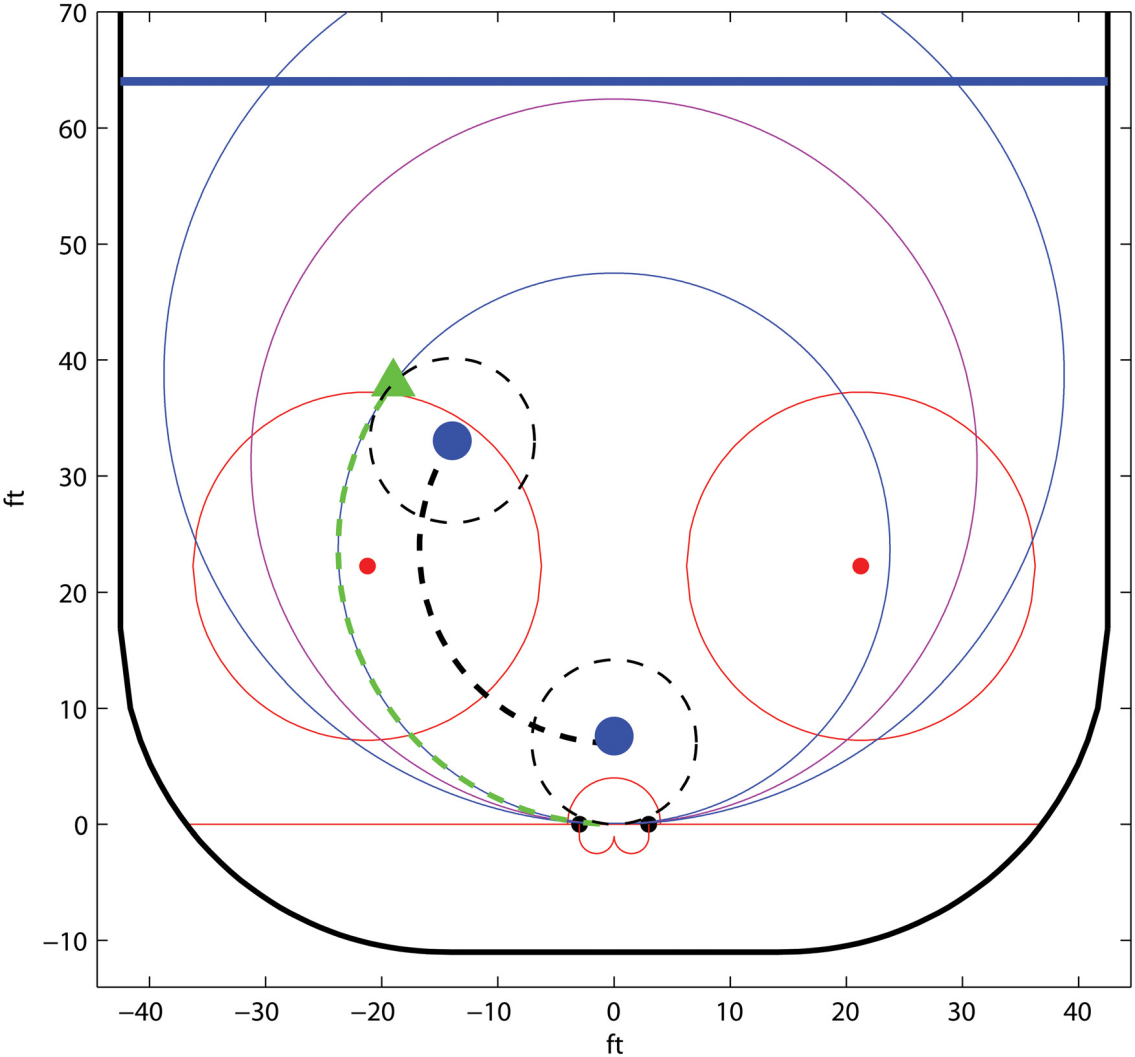
The optimal *B*_1_ strategy for the example *Ice hockey; eccentric isolines*. The offensive starting position is denoted by a triangle and the defensive positions are denoted by circles. Here *B*_1_ has critical speed $v_{B}=\frac{v_{A}\left(r_{A}-r\right)}{r_{A}}$ and the present isoline is circular.

Consider a one against one situation in ice hockey. We assume that the team A attacker *A*_1_ is skating towards the goal with speed *v*_*A*_. The attacker *A*_1_ can not shoot if she is closer than *r* to the team B defender *B*_1_, since *B*_1_ can then interfere with the shot. Further, if the attacker *A*_1_ decides to try to skate past *B*_1_ outside her reach, and the isoline which she is presently skating along is circular with radius *r*_*A*_, what is the necessary speed *v*_*B*_ of *B*_1_ that can guarantee that *A*_1_ will not be able to obtain a higher shot potential before reaching the goal? We see immediately that *v*_*B*_ must satisfy

$$\begin{array}{c} \frac{v_{A}}{v_{B}}\leq \frac{r_{A}}{r_{A}-r}, \end{array}$$

so that $v_{B}\geq \frac{v_{A}\left(r_{A}-r\right)}{r_{A}}$. If *B*_1_ can keep this critical speed, then she should attempt to hold *A*_1_ at the present isoline. If not, *B*_1_ should fall back and hold an isoline associated with a larger shot potential, see Fig 7.

**Remark 23** *Note that the team B strategy in the example above minimizes the maximal shot potential during the course of the situation.*

#### Ice hockey; parametric isolines

Beside the skills of the shooter, the probability of scoring with a shot in any team sport depends on at least two factors; the distance to the goal and the firing angle. Obviously, the chance of scoring will improve the closer to the goal, and the more central the position, the shot is fired from.

We derive here two simple parametric models of the shot potential. The first one is for a team A puck holder. The second is for another team A player who does not have puck possession and who shoots instantly when she get the puck—a so called one timer. We define the shot potential model for the puck holder as

$$\begin{array}{c} f(x_{1})=\alpha e^{-\lambda r_{1}}\cos\left(\theta_{1}\right), \end{array}$$

and the shot potential for the player who shoots a one timer as

$$\begin{array}{c} g(x_{1},x_{2})=(\alpha +\beta |\theta_{1}-\theta_{2}|)e^{-\lambda r_{2}}\cos(\theta_{2}). \end{array}$$

**Fig 8:**
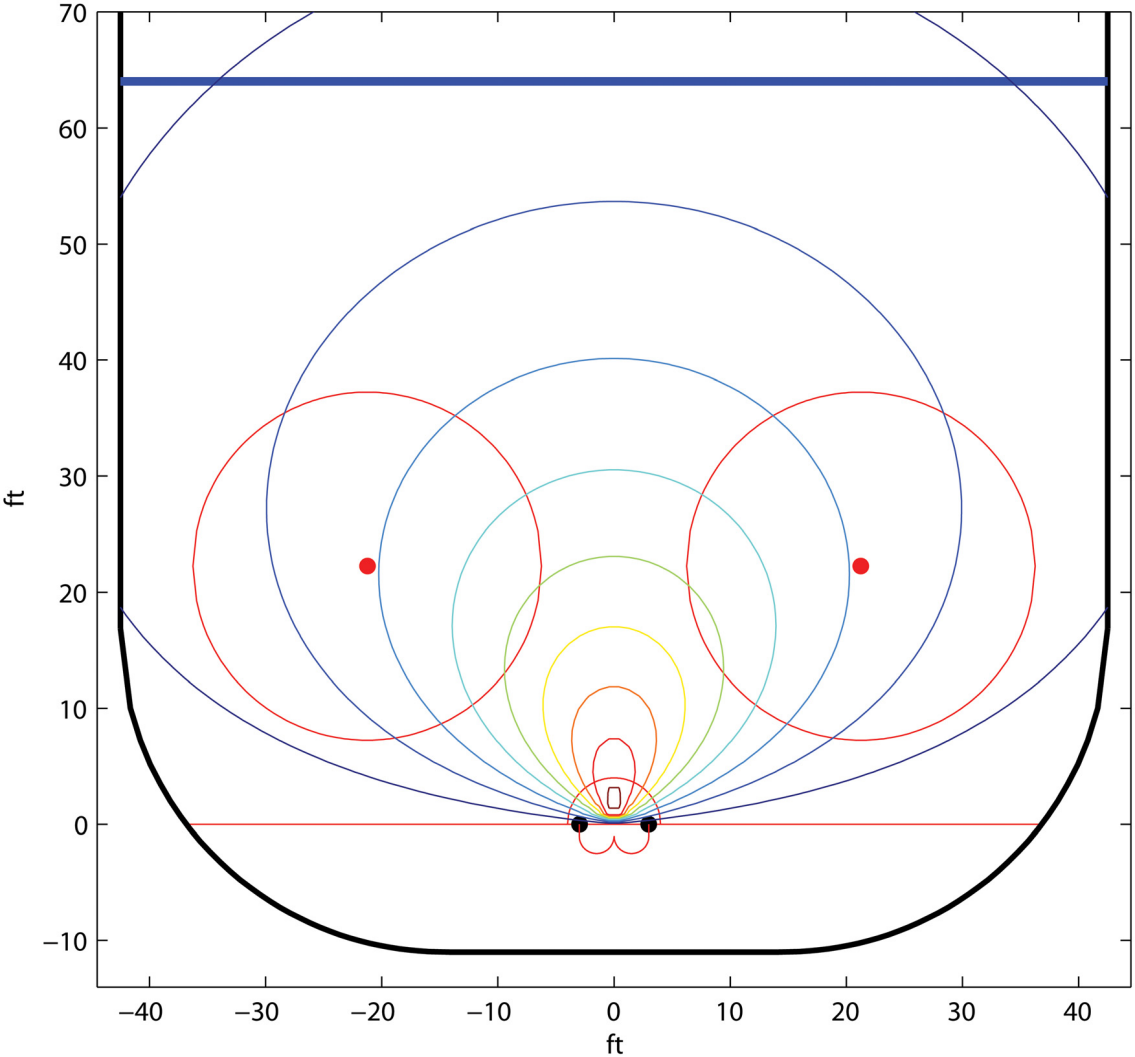
The isolines of the shot potential of *A*_1_ in the example *Ice hockey; parametric isolines*. The parameter values *λ* = 0.03, *α* = 0.2.

**Fig 9:**
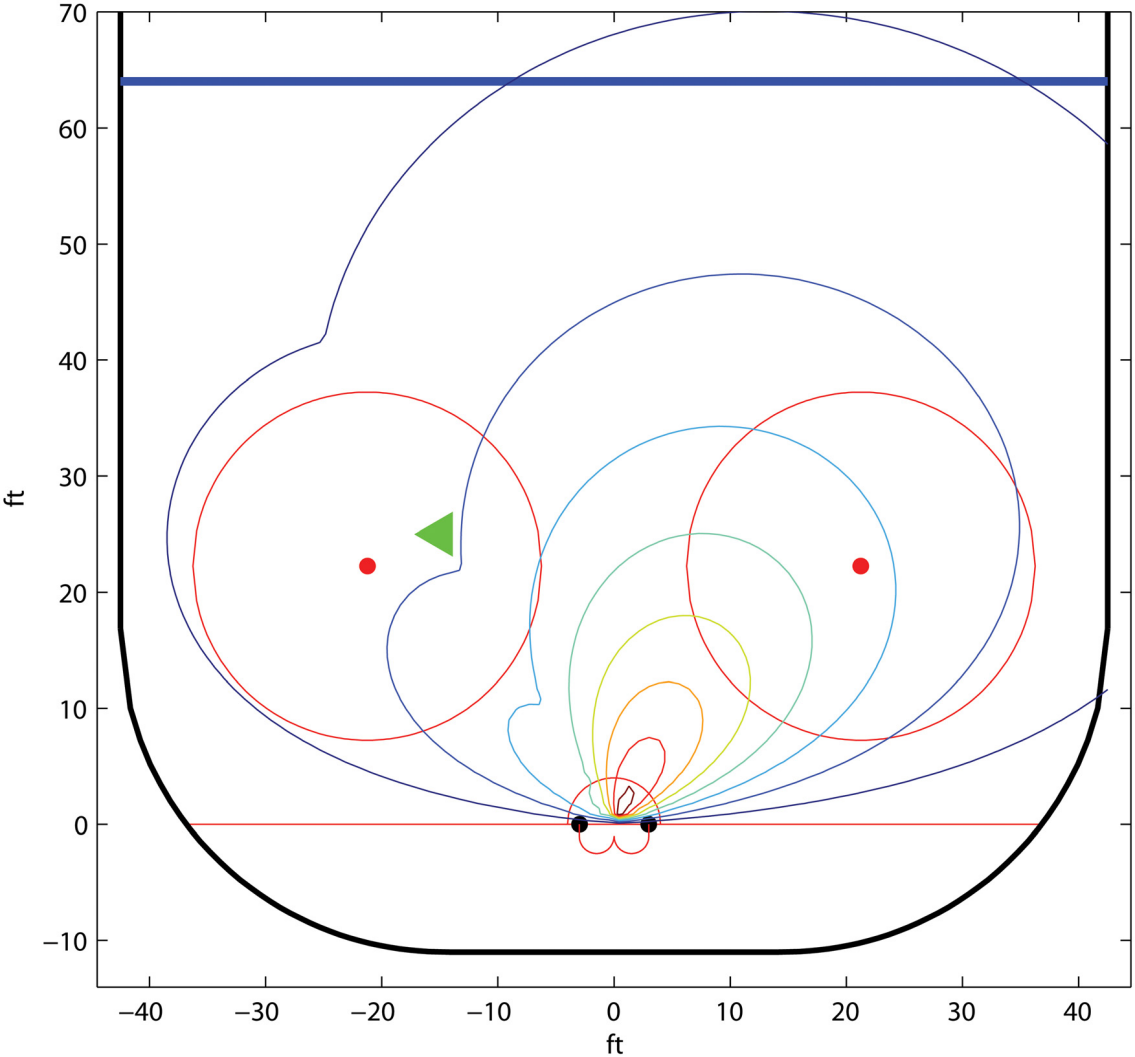
The isolines of the shot potential of *A*_2_, given that the pass comes from *A*_1_ in the example *Ice hockey; parametric isolines*. The assisting player *A*_1_ is positioned at the green triangle and the parameter values are *λ* = 0.03, *α* = *β* = 0.2.

Here *x*_*i*_, *i* = 1, 2, are the locations for *A*_*i*_, (*r*_*i*_, *θ*_*i*_) is the polar representation of *x*_*i*_ around the y-axis for a coordinate system with origo in the middle of the goal, *λ*, *α*, *β* > 0, and *α* + *βπ* ≤ 1. The players’ shot potentials are illustrated in Figs 8 and 9.

The motivation for the second model is that a one timer may have a considerably larger shot potential than if that shot would have been fired from a player who has had puck possession for some time. The reason is that, in the one timer case, the goalkeeper needs to make a sudden shift of position, while for a direct shot, the goalkeeper is already well positioned to save the puck as the shot is fired.

#### Ice hockey; optimal trajectories

Here we use the parametric shot potential model defined in Section *Ice hockey; parametric isolines* to derive the optimal trajectories for a simple example of a two player offense facing a single defender. All trajectories are given on a discretized grid. Recall that the game tree of this setting is given in Fig 5. We assume that the *A*_1_ move to dribble, (*p*_1_, *p*_2_), is dominated by the other alternatives. The player *B*_1_ makes the first move.

The positions for the participating players are denoted by $p_{X}^{\left(i,j\right)}$, where *X* denotes the player, *i* is the level starting from the top, and *j* gives the node in that level counted from the left. We assume that *B*_1_ is the center point of a ball with radius *r* and that the offensive players keep at least that distance to *B*_1_ at all nodes. If *B*_1_ is close to the forward who is receiving a pass, then *B*_1_ intercepts that pass with probability *q*_*c*_ ∈ [0, 1]. Otherwise, *B*_1_ intercepts passes with probability *q*_*p*_ ∈ [0, 1], where *q*_*c*_ > *q*_*p*_. The potential of a shot fired by a puck possession holder *X* is given by $f(p_{X}^{\left(i,j\right)})$. Similarly, the shot potential of a one timer from *Y* following a pass from player *X* is $g\left(p_{X}^{\left(i,j\right)},p_{Y}^{\left(k,l\right)}\right)$.

The time dynamic locations for the players may be illustrated in game trees. These trees describe the trajectories for each player, and depend on the moves of *B*_1_. The direct followers to the inner positions of the game tree for each player are listed in Table 4. The ending positions are given in Table 5.

**Table 4:** The direct followers to inner positions, with location given by $p_{X}^{\left(i,j\right)}$, for a player *X*.

Inner positions	Direct followers
$p_{B_{1}}^{\left(0,1\right)}$	$\left\{p_{B_{1}}^{\left(1,1\right)},p_{B_{1}}^{\left(1,2\right)},p_{B_{1}}^{\left(1,3\right)},p_{B_{1}}^{\left(1,4\right)}\right\}$
$p_{B_{1}}^{\left(1,1\right)}$	$\left\{p_{B_{1}}^{\left(2,1\right)},p_{B_{1}}^{\left(2,2\right)},p_{B_{1}}^{\left(2,3\right)}\right\}$
$p_{B_{1}}^{\left(1,2\right)}$	$\left\{p_{B_{1}}^{\left(2,2\right)},p_{B_{1}}^{\left(2,3\right)},p_{B_{1}}^{\left(2,4\right)},p_{B_{1}}^{\left(2,5\right)}\right\}$
$p_{B_{1}}^{\left(1,3\right)}$	$\left\{p_{B_{1}}^{\left(2,3\right)},p_{B_{1}}^{\left(2,4\right)},p_{B_{1}}^{\left(2,5\right)},p_{B_{1}}^{\left(2,6\right)}\right\}$
$p_{B_{1}}^{\left(1,4\right)}$	$\left\{p_{B_{1}}^{\left(2,4\right)},p_{B_{1}}^{\left(2,5\right)},p_{B_{1}}^{\left(2,6\right)}\right\}$
$p_{B_{1}}^{\left(2,4\right)},p_{B_{1}}^{\left(2,5\right)},p_{B_{1}}^{\left(2,6\right)}$	$\left\{p_{B_{1}}^{\left(3,1\right)}\right\}$
$p_{A_{2}}^{\left(0,1\right)}|\left\{p_{B_{1}}^{\left(1,j\right)}\right\}$	$\left\{p_{A_{1}}^{\left(1,j\right)}\right\}$
$p_{A_{1}}^{\left(i,j\right)}$	$\left\{p_{A_{1}}^{\left(i+1,j\right)}\right\},\;i>0$
$p_{A_{2}}^{\left(0,1\right)}$	$\left\{p_{A_{2}}^{\left(1,1\right)}\right\}$
$p_{A_{2}}^{\left(1,1\right)}|\left\{p_{B_{1}}^{\left(2,1\right)},p_{B_{1}}^{\left(2,2\right)},p_{B_{1}}^{\left(2,3\right)},p_{B_{1}}^{\left(2,4\right)}\right\}$	$\left\{p_{A_{2}}^{\left(2,1\right)},p_{A_{2}}^{\left(2,2\right)},p_{A_{2}}^{\left(2,3\right)}\right\}$
$p_{A_{2}}^{\left(1,1\right)}|\left\{p_{B_{1}}^{\left(2,5\right)}\right\}$	$\left\{p_{A_{2}}^{\left(2,2\right)},p_{A_{2}}^{\left(2,3\right)}\right\}$
$p_{A_{2}}^{\left(1,1\right)}|\left\{p_{B_{1}}^{\left(2,6\right)}\right\}$	$\left\{p_{A_{2}}^{\left(2,3\right)}\right\}$
$p_{A_{2}}^{\left(2,1\right)}|\left\{p_{B_{1}}^{\left(2,1\right)},p_{B_{1}}^{\left(2,2\right)},p_{B_{1}}^{\left(2,3\right)}\right\}$	$\left\{p_{A_{2}}^{\left(3,1\right)},p_{A_{2}}^{\left(3,2\right)},p_{A_{2}}^{\left(3,3\right)},p_{A_{2}}^{\left(3,4\right)}\right\}$
$p_{A_{2}}^{\left(2,1\right)}|\left\{p_{B_{1}}^{\left(2,4\right)}\right\}$	$\left\{p_{A_{2}}^{\left(3,2\right)},p_{A_{2}}^{\left(3,3\right)},p_{A_{2}}^{\left(3,4\right)}\right\}$
$p_{A_{2}}^{\left(2,2\right)}$	$\left\{p_{A_{2}}^{\left(3,3\right)},p_{A_{2}}^{\left(3,4\right)}\right\}$
$p_{A_{2}}^{\left(2,3\right)}$	$\left\{p_{A_{2}}^{\left(3,4\right)}\right\}$

**Table 5:** The ending positions for each player’s game tree.

Player	Ending positions
*B*_1_	$p_{B_{1}}^{\left(2,1\right)},p_{B_{1}}^{\left(2,2\right)},p_{B_{1}}^{\left(2,3\right)},p_{B_{1}}^{\left(3,1\right)}$
*A*_1_	$p_{A_{1}}^{\left(3,1\right)},p_{A_{1}}^{\left(3,2\right)},p_{A_{1}}^{\left(3,3\right)},p_{A_{1}}^{\left(3,4\right)}$
*A*_2_	$p_{A_{2}}^{\left(3,1\right)},p_{A_{2}}^{\left(3,2\right)},p_{A_{2}}^{\left(3,3\right)},p_{A_{2}}^{\left(3,4\right)}$

Note that since it is easier for *B*_1_ to intercept a pass if she is close to the pass receiving forward, the optimal ending position for *B*_1_ must always be such that she puts pressure on both offensive players simultaneously. For this reason, this ending position is the only one we will include for *B*_1_ on the final level.

**Fig 10:**
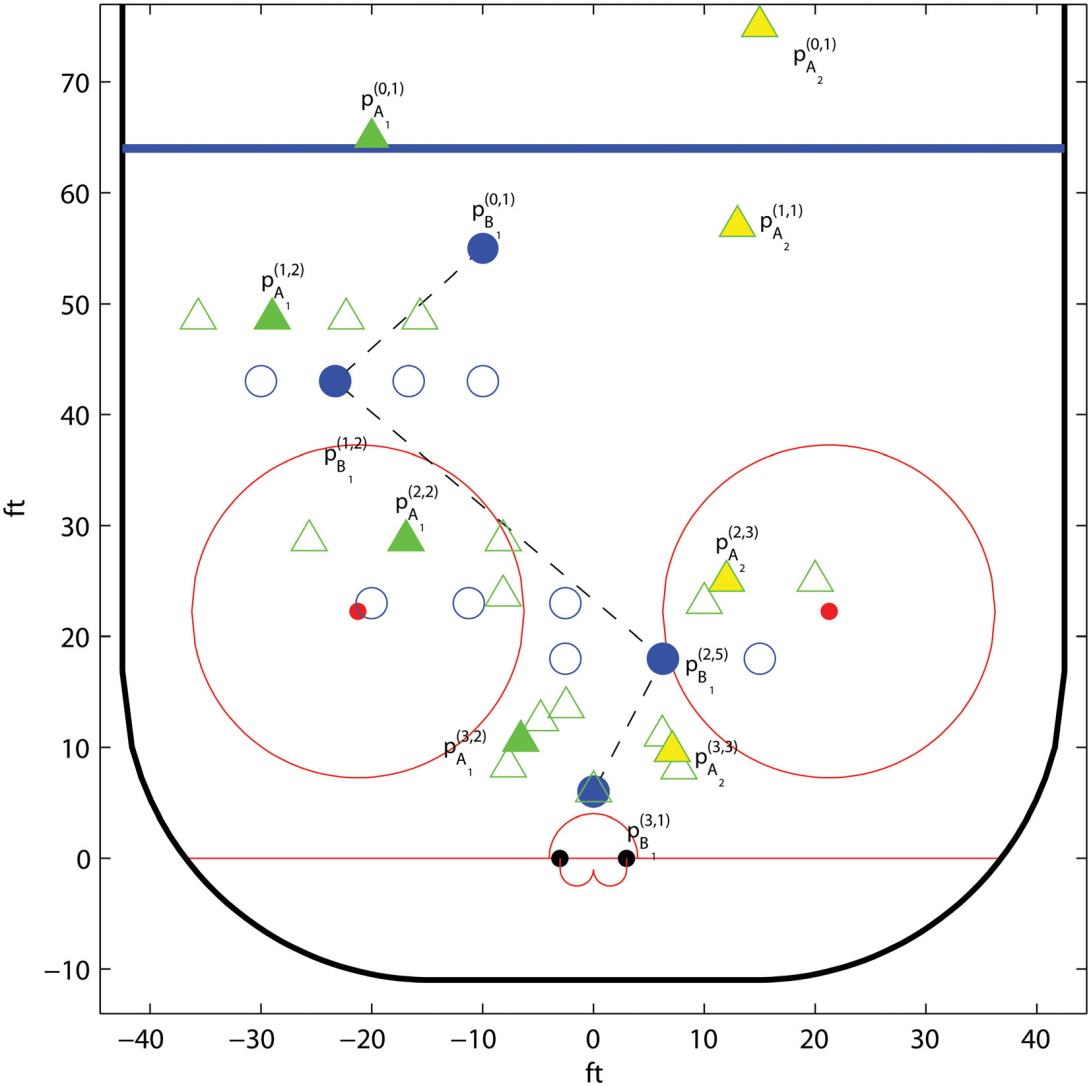
Example of a grid and the corresponding optimal trajectories for *A*_1_, *A*_2_, and *B*_1_, in the example *Ice hockey; two against one*. The offensive players are denoted by triangles, where a solid triangle marks the possession holder, and the defender is denoted by a circle. We use the shot potential model in Section *Ice hockey; parametric isolines* with parameters *α* = *β* = 0.2, *λ* = 0.03, *q*_*p*_ = 0.1, and *q*_*c*_ = 0.5.

The grid for a specific example is given in Fig 10, where the filled circles and triangles indicate the optimal strategies. The trajectory of *A*_1_ depends on how much pressure *B*_1_ puts at level 1. Similarly, the trajectory of *A*_2_ is determined by the position of *B*_1_ at level 2. Note that the optimal strategy is S shaped, which is in line with the reasoning in Section *Ice hockey; two against one*.

**Remark 24** *We have chosen a sparse grid for simplicity of exposition. However, it is straightforward to consider more dense ones. This would allow for more realistic modeling. Further, our results in the example are robust to variations in how the nodes are positioned.*

## Parameter estimation

We have in this paper derived game theoretic models which are general enough to cover a wide range of game situations in several team sports. The applicability of the theory will depend, among other things, on how well we can statistically estimate the model parameters. To this end, we will in this section briefly describe some possible approaches to parameter estimation.

### Statistical analysis of game data

We have in previous sections analyzed a number of game situations which are frequently occurring in their respective sport. By suitably categorizing entire games into a number of such situations, what choices the players made, and the outcome, it is in principle straightforward to obtain estimates of the model parameters for those particular situations. By doing this, one can conclude whether teams as a whole, or even specific players, appear to play in a non-minimax optimal way.

#### One against one

Previously in this paper, we have addressed this game situation in an ice hockey setting. However, it is very common in many other sports as well, including basketball, soccer, and team handball. Given a specific sport, we assume that we have observed a number of games for team *B*. In these games, we have *n* occasions which we have judged to be one against one situations. Further, we categorize the choices that the offensive players did in each situation into either *shoot* or *dribble*. We have now *n*_*s*_ data points where the offensive player chose to shoot, and *n*_*d*_ situations where she decided to dribble. The number of goals scored when the offensive players decided to shoot and dribble is denoted by *X*_*s*_ and *X*_*d*_, respectively. Hence, *X*_*s*_ ∼ Bin(*n*_*s*_, *p*_*s*_) and *X*_*d*_ ∼ Bin(*n*_*d*_, *p*_*d*_). We can now apply standard statistics to draw conclusions. E g, we can investigate whether we can reject the hypothesis that the probability of scoring is the same for the shoot and the dribble alternative, respectively. If we are able to reject this hypothesis, we can also conclude that team *B* does not appear to play the optimal min-max strategy.

### Experimental design

Given a game situation, it is reasonable to aim to find the overall optimal strategy. However, due to the complexity of many game situations, it is likely to be insufficient to merely analyze game data. The reason is that this approach only has in its scope to discover if an existing strategy is better than some other existing strategy. It will be less efficient at determining what is indeed the actual optimal strategy. To be able to obtain this, we need to consider experimental design.

#### Parameterizing the isolines

While we, thus far, have discussed isolines mainly in relation to ice hockey, the concept is central to e g basketball, soccer and team handball as well. The reason is that the isolines are important factors in determining how to defend in one against one, two against one and many other common game situations. To estimate the isolines in a controlled experiment is particularly straightforward. Random players take shots from random positions, in relevant cases at an undisturbed goalkeeper. The outcome (goal or no goal), as well as the position from which the shot was taken, is recorded. To estimate the isolines can now be done by e g applying standard logistic regression techniques.

#### Ice hockey; chasing the puck

It is straightforward to set up a controlled experiment for the example Ice hockey; chasing the puck. In the setting of the experiment, we can for each team *B* defender *B*_1_ run the game situation multiple times. For each situation, *B*_1_ gets assigned if she should either play the alternative to charge or if she should use the other option; to hold back. The offensive player *A*_1_, who is assigned at random for each situation, will always charge, by the analysis in the example Ice hockey; chasing the puck. We can run the experiment a sufficiently large number of times to be able to conclude to what extent either of the defensive alternatives are better than the other. Examples similar to the present one can be found in several other team sports.

## Discussion

In this paper, we make an attempt to develop a mathematical theory on game intelligence in team sports. It is central to this theory to value game situations by their potential. Intuitively, the potential is the probability that the offense scores the next goal minus the probability that the next goal is made by the defense. We give many examples to illustrate the width of the applicability of our results, but the set of chosen situations is by no means exhaustive.

In Section *Team handball; shot proportions*, we argue that the classical efficiency thresholds—e g that back court and wing players should have 50% and 80% mean shot efficiency, respectively—are likely to be non-optimal. It would be interesting to investigate this issue further.

One of the authors, Nicklas Lidström, relied on a set of first principles which he used to analyze how to play game situations during his career as a professional ice hockey player. His approach constitutes a cornerstone in the present paper.

In Section *Ice hockey; chasing the puck*, we analyzed whether a defense player should charge or hold back. We note here that it appears that the vast majority of backchecking hockey players judge that it is optimal to charge. Nicklas thought that it was optimal for him to hold back, which consequently was how he played in such situations.

The example in Section *Ice hockey; pass or dribble* presents Nicklas’ analysis of why it is optimal to pass early in that situation, and in similar ones.

Further, in one against one situations, Nicklas recalls using his reached out stick extensively as a first line of defense against opposing forwards. He believed that this increased the width with which he could operate. The operating width is equivalent to the quantity *r* in the examples of this paper. Hence he made the action to dribble less attractive in terms of potential for the opposing forwards. This had the effect that he could stear the forward further to the sides—preferably their back hand side—to make that alternative, too, lower in potential than what he thought he could have obtained otherwise.

We note that Nicklas’ S shaped strategy in the two against one examples in ice hockey is different to how most defense players choose to play such situations. It seems that most defenders eventually decide to let go of the non-puck holding forward to focus on the forward with puck possession instead.
